# Indoor location perception model based on Resnet50 and Elman network

**DOI:** 10.1371/journal.pone.0338316

**Published:** 2025-12-22

**Authors:** Pengjun Zhang, Jie Mi

**Affiliations:** 1 School of Architectural Engineering, Hebei Vocational University of Industry and Technology, Shijiazhuang, China; 2 Department of Logistics Management, Hebei Jiaotong Vocational and Technical College, Shijiazhuang, China; Istinye University: Istinye Universitesi, TÜRKIYE

## Abstract

The visible light indoor position perception method not only solves the limitations of traditional positioning technology indoors, but also promotes innovation in fields such as smart retail and healthcare with its advantages of high accuracy and low cost. At present, visible light indoor positioning methods based on received signal strength are restricted by issues such as environmental interference and poor signal stability. Additionally, traditional feature extraction methods result in insufficient diversity in feature databases, and the initial parameters of positioning models tend to fall into local optima, leading to significant fluctuations in positioning errors in complex indoor scenarios and making it difficult to consistently achieve high-precision positioning results. Therefore, it is necessary to address the problems of weak signal anti-interference capability, inadequate feature representation, and insufficient model parameter optimization by focusing on three core aspects: data collection, feature extraction, and model optimization, thereby providing a technical pathway to enhance the accuracy and stability of indoor positioning. In this regard, to improve the stability and accuracy of this technology, the design of light source layout and image data acquisition method was studied. This research developed an image data feature extraction method based on Resnet50 and referenced the idea of feature pyramid. Meanwhile, an indoor location sensing model based on Elman network was designed, and an improved grey wolf optimization algorithm was proposed to optimize the initial weights and threshold parameters of the Elman network. The results showed that the average cosine similaritycorresponding to the feature extraction methoddesigned in the studywas 0.103, which wascloser to 0, indicating that the feature library constructed by this method was more diverse. Under different test functions, the average fitness values of the improved grey wolf optimization algorithm were 2.69 × 105, 1.47 × 101, and 2.17 × 102, respectively, all of which were lower than the comparison algorithm. At different heights, the average errors of the designed perception model were 3.04 cm, 3.57 cm, and 3.19 cm, respectively, which were notably lower than the comparison models. The design model also performed better in dynamic analysis and environmental light impact analysis. The designed perception model exhibited excellent performance and is capable of offering technical assistance for improving indoor positioning accuracy.

## 1. Introduction

In today’s digital age, indoor location sensing technology is gradually becoming a key force driving the development of fields such as smart living, intelligent transportation, and smart healthcare [1,2].With the increasing demand for precise positioning, traditional indoor positioning approaches can no longer fulfill the intricate demands of modern application scenarios. For example, the Global Positioning System and Beidou have signal attenuation due to wall obstruction, which cannot meet the precise requirements of indoor positioning. Wireless local area networks and Bluetooth are susceptible to environmental interference, while ultra wideband is expensive and has limited coverage. Infrared radiation is also susceptible to heat source interference. These limitations make it difficult for traditional indoor positioning methods to adapt to modern complex and ever-changing indoor positioning scenarios. In this context, indoor position sensing technology based on visible light communication has emerged. It not only overcomes many limitations of traditional positioning technology indoors, but also brings unprecedented innovation opportunities to many fields such as smart retail and healthcare with its significant advantages of high accuracy, low cost, and green environmental protection [3–5]. Indoor position sensing technology based on visible light communication combines lighting and positioning functions, utilizing visible light band communication of light emitting diodes (LEDs) to completely avoid electromagnetic interference. And this technology can achieve precise signal coverage and reduce interference by adjusting the illumination angle and intensity of LED lights. In addition, this technology combines high data transmission rates with multiple positioning methods, such as ranging and intersection methods. By receiving signal strengths or unique identifiers from multiple LED light sources and combining them with corresponding algorithms, it can achieve sub-meter or even centimeter level high-precision positioning. However, despite the enormous development potential of visible light indoor position sensing technology, the current perception methods based on received signal strength still suffer from poor stability, which to some extent limits their widespread application. Regarding this issue, commonly-used methods include multi-point localization, fingerprint-based localization, and improved particle filtering algorithms [6,7].

However, these methods and studies also have certain shortcomings. For example, the multi-point positioning method has low positioning accuracy in indoor edge areas, and fingerprint-based positioning methods require frequent updates to the fingerprint database. The essence of visible light image data is the extension and visualization of the received light intensity in the spatial dimension. Specifically, the grayscale value of each pixel in the image corresponds to the received light intensity at its spatial position, and the entire image presents the complete distribution of light intensity in indoor space. It can be said that describing the signal model from the perspective of received light intensity is the physical basis for constructing visible light image data; Visible light image data, on the other hand, is an extended form of received light intensity from “single point scalar” to “spatial distribution”. To tackle the stability and accuracy challenges of the received signal strength perception method, a feature extraction method based on Resnet50 was proposed, a feature library was constructed, and an indoor position perception model based on Improved Grey Wolf Optimization (IGWO) optimized Elman network was designed. The motivation for research is to improve the stability and accuracy of visible light indoor position sensing technology based on received signal strength, and to solve the problem of poor stability in practical applications. The research aims to construct a high-performance indoor location perception model, providing reliable technical support for improving indoor positioning accuracy and assisting in the construction and development of a smart society. The innovation of the research lies in the combination of data collection, feature extraction, and model construction, and the introduction of IGWO to optimize the initial weights and thresholds of Elman networks, enhancing the stability and accuracy of indoor position perception technology.

There are a total of three contributions to the research. Firstly, innovative solutions have been proposed to address the limitations of existing visible light indoor positioning technologies. The existing visible light positioning methods based on received signal strength have shortcomings in terms of stability and accuracy. However, research has effectively improved the stability and accuracy of positioning by combining Resnet50 feature extraction and IGWO, solving the performance bottleneck problem of traditional methods in complex environments. Secondly, the research has expanded the theoretical and practical boundaries of visible light indoor positioning technology. By introducing deep learning feature extraction and optimization algorithms, not only has the positioning accuracy been improved, but the robustness and environmental adaptability of the system have also been enhanced. This combination of computer vision and deep learning provides new research ideas and technological paths for the field of indoor positioning, promoting interdisciplinary integration. Finally, the research has a significant driving effect on practical applications. By improving positioning accuracy and stability, this study provides more reliable technical support for indoor positioning applications in smart retail, healthcare, and other fields, which helps to enhance user experience and operational efficiency, and has important practical application value.

The structure of the sections is arranged as follows: section 1 is an introduction, systematically elaborating on the research background and significance of indoor position perception technology, and proposing the motivation, objectives, innovation points, and main contributions of this study, laying a theoretical and directional foundation for subsequent research. Section 2 is related work, which summarizes the research results in the field of visible light indoor positioning in recent years and clarifies the limitations of existing research. Section 3 is about methods and materials, which elaborates on data collection design, feature extraction method based on Resnet50, and the construction process of indoor position perception model optimized by IGWO Elman network. Section 4 presents the experimental results, which quantitatively validate the performance advantages of the proposed model through algorithm performance verification and case analysis. Section 5 discusses and analyzes the limitations and potential threats of the research. Section 6 is the conclusion and future work, summarizing the core research findings. Section 7 is about limitation and future work, further refining future research directions.

## 2. Related work

In the field of indoor positioning, visible light positioning technology has received widespread attention due to its advantages of high accuracy, low cost, and green environmental protection. Numerous scholars have conducted in-depth research on this technology, dedicated to improving positioning accuracy and stability. Jia C and other researchers proposed a high-precision 3D indoor visible light positioning method based on an improved adaptive cuckoo search algorithm to alleviate the error caused by the rotation of photodiodes in visible light positioning systems. The method adopted an optical channel transmission model and set the indoor space size to 5m × 5m × 6m. The results showed that the average 3D positioning error was 16.48 cm when the photodiode rotated [8]. Liu X et al. designed an enhanced visual object tracking algorithm tto achieve a more harmonious equilibrium among positioning precision, real-time capabilities, and resilience in visible light positioning systems. This method included a lightweight recognition/demodulation mechanism and utilized Kalman filtering, as well as Gaussian mixture models. The results showed that this method could achieve high-precision dynamic positioning and improve the overall performance of the system [9]. Lee G et al. designed a passive shadow-based passive visible light localization system to address the issue of only recognizing approximate shadows, and defined its mathematical model. The outcomes indicated that the system achieved an average positioning error of 7.6 cm within a 0.4 × 0.25m positioning area with a height of 1.7m [10]. Zhu R et al. proposed a data preprocessing method including data cleaning and data augmentation to address the dependence of visible light positioning technology based on received signal strength on fingerprint density, with the aim of minimizing the impact of noisy samples. The outcomes indicated that the average positioning error of this method was 1.7 cm, and it was also feasible on resource limited embedded devices [11]. Yang X et al. proposed a positioning framework containing a proportional model to improve the positioning precision and robustness of visible light positioning systems based on photodiodes, and designed new parameters to connect the received signal strength ratio with its corresponding distance ratio. The results showed that in scenes affected by ambient light, the localization accuracy of this method was improved by more than 50% compared to the traditional Lambert model [12]. Shi J and other experts have designed a new positioning scheme to address the challenge of visible light positioning systems relying on multiple LEDs with a single light source available. The intelligent reflector is used as a virtual light source for visible light positioning, and a new positioning scheme is proposed to reduce spatial interference from multiple intelligent reflectors and accurately estimate their power. The results showed that the scheme achieved a positioning accuracy of 0.056m under 16 intelligent reflective surface units [13]. In order to alleviate the negative impact of dust on visible light positioning technology based on received signal strength, scholars such as Jin L modified the classic Lambertian based channel model and proposed a two-point method for channel model calibration based on two received signal strength measurements. They further designed a single point method, where each channel model calibration only requires one received signal strength measurement. The results showed that the average positioning error remained below 6 cm in different dust accumulation scenarios [14].

The comprehensive summary of different literature is shown in Table 1.

**Table 1:** Comprehensive summary table of different literature.

Average error	Maximum positioning error	Computational complexity	Average time consumption	Reference number
16.48 cm	18.59 cm	High	162ms	[8]
4.90 cm	8.37 cm	High	149ms	[9]
7.60 cm	11.98 cm	Medium	187ms	[10]
1.75 cm	4.02 cm	Medium	95ms	[11]
3.97 cm	5.68 cm	High	132ms	[12]
2.80 cm	3.01 cm	Medium	72ms	[13]
5.89 cm	6.33 cm	Medium	88ms	[14]

Based on existing research, it can be seen that there are still three urgent issues that need to be addressed in visible light indoor positioning technology: firstly, the feature extraction dimension is single, and existing methods rely more on traditional signal features or shallow image features, which do not fully explore the deep semantic information of the image, resulting in insufficient diversity in the feature library and difficulty in supporting high-precision positioning. The second issue is the insufficient optimization of model parameters, which means that traditional optimization algorithms are prone to getting stuck in local optima, making it difficult to further reduce positioning errors. Thirdly, the environmental adaptability is limited, and most studies show significant performance degradation under environmental light interference or dynamic scenes, making it difficult to meet the requirements of complex practical scenarios.

The research design method fills the above gaps through three innovative aspects, and the performance advantages can be quantified: 1 In terms of feature extraction, the feature library constructed based on Resnet50’s feature extraction method has an average cosine similarity of 0.103, which is much lower than Inception v3’s 0.845 and VGG-16’s 0.801, indicating a significant improvement in feature diversity. In terms of model optimization, the average fitness values of the IGWO algorithm on different test functions are lower than those of the comparison algorithm, allowing the Elman network to avoid local optima. In terms of environmental adaptability, the maximum errors of the model in this article are 23.87 cm and 21.09 cm in the absence and presence of external light, respectively, and the positioning accuracy is better in multi story building applications.

## 3. Methods and materials

To deal with the issue of improving the accuracy of visible light indoor position perception methods, a research was conducted on the design of image data acquisition methods and the explanation of data preprocessing methods. Subsequently, a Resnet50-based image data feature extraction method was developed, and an indoor position perception model based on Elman network was designed.

### 3.1. Data acquisition design considering light source layout and bilinear interpolation method

In the fields of smart retail and healthcare, users’ demand for indoor positioning technology is mainly reflected in high precision, low cost, and real-time performance. Based on this user demand, the study fully considered various technical options when designing an indoor location perception model. After comprehensive evaluation of the advantages of high precision and low cost of visible light communication technology, as well as the limitations of traditional positioning technology, visible light communication technology was ultimately selected as the research foundation.

To improve the accuracy of visible light indoor position perception methods, the entire technical process was systematically designed. Firstly, the study conducted light source layout and visible light image acquisition. The layout of light sources is the foundation of indoor positioning, and a reasonable layout can ensure uniform coverage and effective reception of signals. Therefore, the study conducted in-depth analysis and comparison of various light source layout schemes, selected the optimal design, and based on this, collected visible light images. Secondly, a feature extraction model based on Resnet50 was designed, and the additive fusion method was used for deep feature fusion. Based on the powerful feature extraction capability of Resnet50, research can extract rich depth features from collected visible light images and provide high-quality information foundation for the subsequent construction of indoor position perception models. Meanwhile, by introducing the additive fusion method, the expressive power of features can be further enhanced. Finally, based on the obtained features, an indoor position perception model was constructed using the IGWO algorithm to optimize the Elman network. Through Elman networks, nonlinear transformations and time series processing can be applied to input features, enabling the capture of dynamic changes and temporal dependencies in indoor location perception. The IGWO algorithm includes three improvements: chaos initialization, improved convergence factor, and chaos disturbance strategy.

The research focuses on the positioning problem of a specific indoor area equipped with multiple LED lights. The goal of the research is to accurately determine the position of the receiver based on the received visible light signal. The following is the mathematical description of the problem: Suppose d LED lights are deployed in a three-dimensional indoor space b, and the position of each LED light i is $p_{i}=\left(xi,yi,zi\right)$, with an emitted light intensity of ni. The receiver is located at an unknown position $p_{g}=\left(xg,yg,zg\right)$ to collect visible light image data. So the localization problem is to estimate $p_{g}$ based on collected image data and known LED positions. To this end, the research considers the following factors: Firstly, the light intensity received by the receiver from each LED decreases with the square of the distance between the receiver and the LED. Secondly, the received light intensity is also affected by the incident angle and the characteristics of the surrounding environment. Therefore, the relationship between received light intensity and distance is modeled as shown in equation (1).


$$n_{\Gamma }=n_{i}\cdot \frac{\text{1}}{\psi_{ig}^{2}}\cdot \cos\varphi$$ (1)

In equation (1), $n_{\Gamma }$ is the received light intensity, $\psi_{ig}$ is the distance between LED i and the receiver, and φ is the incident angle. Based on this model, indoor positioning problems can be transformed into a type of optimization problem. Specifically, the research seeks the receiver position $p_{g}$ that minimizes the sum of squared differences between the measured light intensity and the predicted light intensity based on the LED lamp position and model. The expression of this optimization problem is shown in equation (2).


$$\begin{array}{c} \min p_{g}=\mathrm{\backslash arg}\min_{p_{g}}\sum {\mathrm{\backslash nolimits}}_{i=1}^{d}{\left(n_{\Gamma ,i}-n_{i}\cdot \frac{\text{1}}{\psi_{ig}^{2}}\cdot \cos\varphi_{i}\right)}^{2} \end{array}$$ (2)

The difficulty of this optimization problem mainly lies in the need to process a large amount of image data to extract useful features, and accurately identify LED signals from noise and interference. Traditional signal processing methods often fall short in handling such complex data, making it difficult to achieve high-precision positioning requirements. Machine learning methods, especially deep learning, have been proven to have significant advantages in processing complex datasets and high-dimensional data due to their powerful feature extraction and pattern recognition capabilities.

**Fig 1:**
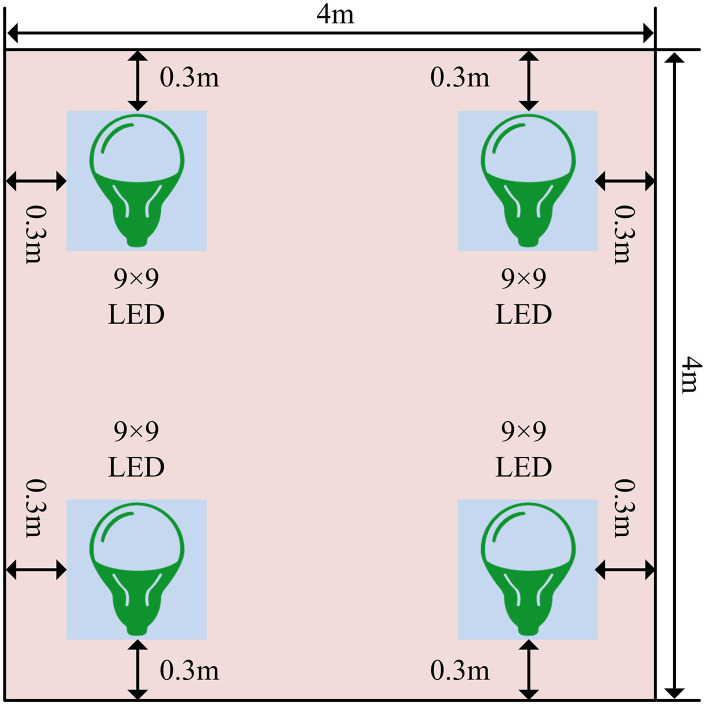
Specific schematic diagram of the layout of four LED arrays.

After problem description and mathematical modeling, the research will proceed with data collection design.In the indoor environment, the study first constructed an indoor model with a size of 4m × 4m × 3m.When conducting visible light indoor positioning, a set of light sources were placed in the indoor environment, and visual sensors determined the position by receiving “visible light image data”. Secondly, in the layout of the light source, three classic layout schemes were referred to, corresponding to 3, 4, and 5 LED array layouts, respectively, and the uniformity under these three schemes was analyzed [15,16]. Finally, four LED layouts were selected for the study, as shown in Fig 1.

**Fig 2:**
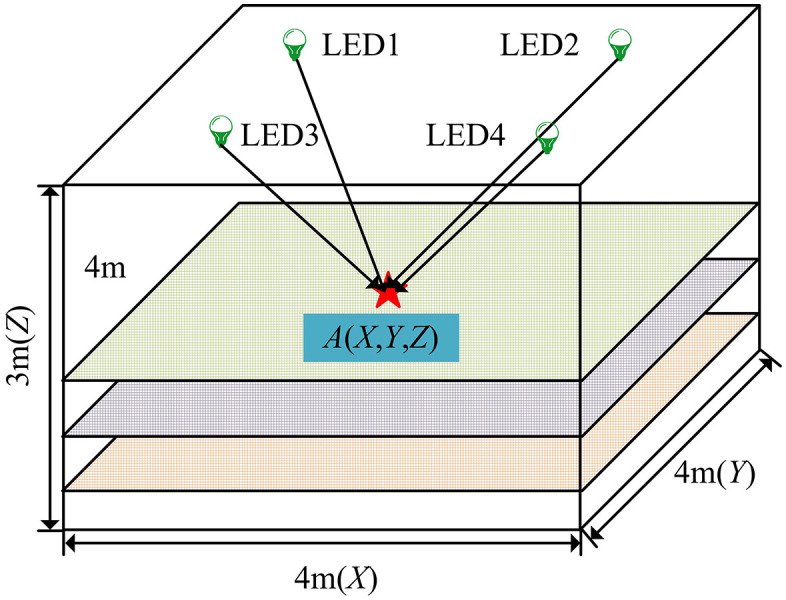
Visible light indoor positioning model.

From Fig 1, there are a total of 4 LEDs in the light source layout, and each LED is in a 9 × 9 format. This design can increase the detail information of the light signal, making the image data received by the visual sensor more abundant, thereby improving the positioning accuracy. In addition, the distance between each LED and the wall is 0.3m. To obtain visible light image data, a visible light indoor positioning model was constructed, as presented in Fig 2.

From Fig 2, during data collection, the study segmented the horizontal and vertical planes of the indoor model at a distance of 0.1m, and segmented the vertical plane at a distance of 0.6m.Therefore, the coordinate of the collection point is A(X,Y,Z). Meanwhile, to maintain data diversity, the study set three collection heights, namely 0m, 0.6m, and 1.2m. Afterwards, data transmission was carried out using a universal serial bus. In addition, it is reasonable to consider LED lights as a single point light source in research. Firstly, the research focuses on verifying the effectiveness of indoor position perception models based on Resnet50 and Elman networks, rather than accurately simulating the physical characteristics of each LED. Simplifying to a point light source helps to focus on exploring the impact of light source layout on positioning accuracy. Secondly, the experiment used LED lights of the same model and maintained the same luminous intensity, ensuring consistency in luminous characteristics and making it feasible to ignore the effects of size and model differences. Furthermore, the receiver receives a composite signal from multiple LED lights, and the signal strength is mainly affected by distance and angle. The size and model of the LED lights have a relatively small impact on the macroscopic distribution of the signal. Finally, in practical applications, more attention is paid to the impact of the overall light source layout on positioning accuracy and stability, rather than the detailed optical characteristics of individual LEDs. Therefore, treating LED lights as point light sources can simplify system design, reduce computational complexity, and improve the real-time and practicality of the system. The data being transmitted is visible light image data collected through visual sensors in the experimental environment. The visual sensor (camera) actually captures a direct image of the LED light itself. The grayscale images directly imaged by these LED lights themselves have the following characteristics in addition to the core acquisition parameters: the four LED spots in the image are symmetrically distributed in a 2 × 2 pattern, with a single spot diameter of about 25–30 pixels at a resolution of 1280 × 720, and the grayscale values of the spots decay in a gradient from the center to the edge, without obvious distortion or overexposure. The purpose of transmission is to transfer the collected data to a computer or other processing device for data processing and analysis, in order to train and optimize indoor positioning models. Due to the large size of the collected data and the involvement of a lot of insignificant information, preprocessing is necessary for the research. Bilinear interpolation is a commonly used interpolation algorithm in numerical analysis, whose main concept involves conducting linear interpolation independently in two different directions [17,18]. Specifically, If one wishes to determine the value of an undefined function f at a point P=(x,y), the values of function f at four points $Q_{11}=\left(x_{1},y_{1}\right)$, $Q_{12}=\left(x_{1},y_{2}\right)$, $Q_{21}=\left(x_{2},y_{1}\right)$, and $Q_{22}=\left(x_{2},y_{2}\right)$ need to be known. Afterwards, linear interpolations in the x-direction andy-direction are performed, and the interpolation results are combined in both directions. Among them, the linear interpolation expression in the x-direction is shown in equation (3) [19].


$$\{\begin{array}{c} {}*20cf\left(x,y_{1}\right)=\frac{x_{2}-x}{x_{2}-x_{1}}\cdot f\left(x_{1},y_{1}\right)+\frac{x-x_{1}}{x_{2}-x_{1}}\cdot f\left(x_{2},y_{1}\right)\\ f\left(x,y_{2}\right)=\frac{x_{2}-x}{x_{2}-x_{1}}\cdot f\left(x_{1},y_{2}\right)+\frac{x-x_{1}}{x_{2}-x_{1}}\cdot f\left(x_{2},y_{2}\right) \end{array}$$ (3)

The y-direction linear interpolation expression is shown in equation (4) [20].


$$f\left(x,y\right)=\frac{y_{2}-y}{y_{2}-y_{1}}\cdot f\left(x,y_{1}\right)+\frac{y-y_{1}}{y_{2}-y_{1}}\cdot f\left(x,y_{2}\right)$$ (4)

The interpolation results in both directions are combined as presented in equation (5).


$$f\left(x,y\right)=\;\frac{f\left(x_{2},y_{2}\right)\left(x-x_{1}\right)\left(y-y_{1}\right)+f\left(x_{2},y_{1}\right)\left(x-x_{1}\right)\left(y_{2}-y\right)}{\left(x_{2}-x_{1}\right)\left(y_{2}-y_{1}\right)}\;+\frac{f\left(x_{1},y_{1}\right)\left(x_{2}-x\right)\left(y_{2}-y\right)+f\left(x_{1},y_{2}\right)\left(x_{2}-x\right)\left(y-y_{1}\right)}{\left(x_{2}-x_{1}\right)\left(y_{2}-y_{1}\right)}$$ (5)

Assuming that the values of $\left(x_{2}-x_{1}\right)$ and $\left(y_{2}-y_{1}\right)$ are both 1, equation (5) can be simplified as shown in equation (6).


$$f\left(x,y\right)=\;f\left(x_{1},y_{1}\right)\left(x_{2}-x\right)\left(y_{2}-y\right)+f\left(x_{2},y_{1}\right)\left(x-x_{1}\right)\left(y_{2}-y\right)\;+f\left(x_{1},y_{2}\right)\left(x_{2}-x\right)\left(y-y_{1}\right)+f\left(x_{2},y_{2}\right)\left(x-x_{1}\right)\left(y-y_{1}\right)$$ (6)

In addition, the bilinear interpolation method considers four neighboring pixels around the target pixel, enabling it to provide more accurate interpolation results and is suitable for various transformations with strong scalability. Therefore, the study utilized bilinear interpolation to preprocess the collected image data, in order to effectively remove redundant information from the data, improve data quality, and lay a solid foundation for subsequent feature extraction and model construction.

### 3.2. Feature acquisition design based on Resnet50

**Fig 3:**
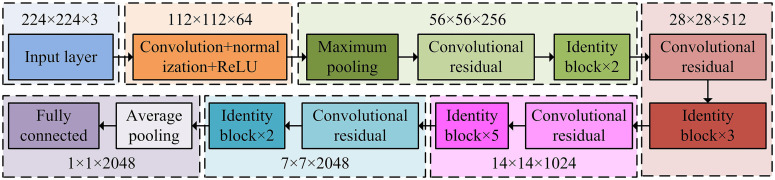
The specific structure of Resnet50.

The researchprovidesa detailed design and explanation of the data collection process, with a focus on exploring the application of bilinear interpolation in image preprocessing. These collected data can provide a high-quality data foundation for the construction of the entire indoor position perception model. In addition, the preprocessed image data will first be input into a Resnet50-based feature extraction model to extract features with rich semantic information, which will be used for the construction of subsequent indoor position perception models. Meanwhile, the input data format of ResNet50 is preprocessed visible light grayscale images. Therefore, based on the processed data, the study will further explore the design of feature acquisition based on Resnet50 and how to use these features to construct an efficient indoor location perception model. In the fields of image processing and computer vision, using pre-trained networks to extract image features is a common method [21,22]. Generally speaking, pre-trained networks have been trained on large-scale datasets and are able to learn rich feature representations, resulting in excellent performance in various tasks.Resnet50 is a deep residual network mainly applied to computer vision functions like categorizing images and identifying objects. The core innovation of ResNet50 lies in its introduction of residual learning framework, which solves the problems of gradient vanishing and exploding in deep network training by skipping connections [23,24]. This network structure enables Resnet50 to maintain high accuracy while having good generalization ability. The specific structure of Resnet50 is presented in Fig 3.

From Fig 3, ResNet50 primarily consists of input layers, max pooling, convolutional residuals, average pooling, and fully connected layers. Through ResNet50, research can extract deep global features from image data, such as global average pooling features. To fully leverage these features, the study employed an additive fusion method for feature fusion. The core idea of the additive fusion method is to add multiple feature maps element-wise to obtain a fused feature map. The advantages of this method include simple implementation, high computational efficiency, and the ability to enhance feature expression [25]. The expression of the additive fusion method is shown in equation (7) [26].


$$B=D_{1}+D_{2}+D_{3}+......+D_{C}=\sum_{C}D_{C}$$ (7)

In equation (7), B represents the fused feature map, $D_{1}$, $D_{2}$, and $D_{C}$ represent the 1st, 2nd, and C th feature maps respectively, and $\sum_{C}D_{C}$ indicates the summation operation for all feature maps. When using this method, it is necessary to maintain that all feature maps have the same size. Therefore, the study adopted pooling operation to adjust the size of the feature map and ensured the same number of channels through convolution operation. At this point, the expression of the additive fusion method has undergone changes and adjustments, and the adjusted content is shown in equation (8).


$$\begin{array}{cc} B & =\left[pool_{1}\left(conv_{1}\left({\hat{D}}_{1}\right)\right),pool_{2}\left(conv_{2}\left({\hat{D}}_{2}\right)\right),...,pool_{C}\left(conv_{C}\left({\hat{D}}_{C}\right)\right)\right]\\ & =\underset{C}{⋃}pool_{C}\left(conv_{C}\left({\hat{D}}_{C}\right)\right) \end{array}$$ (8)

**Fig 4:**
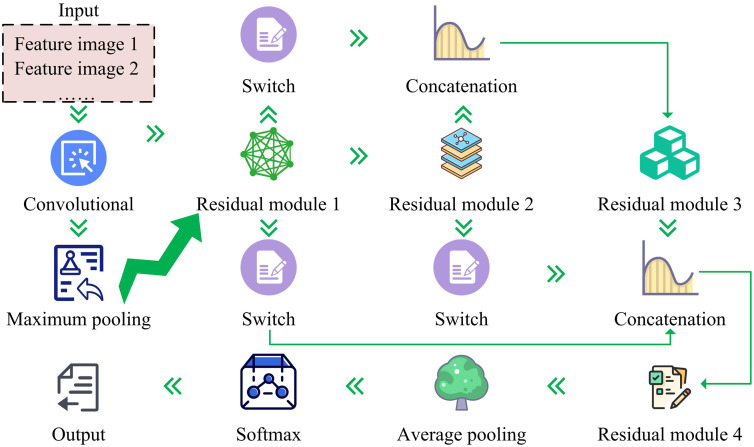
The specific structure of Resnet50.

In equation (8), ${\hat{D}}_{C}$ is the feature, $conv_{C}\left(\cdot \right)$ represents the convolution operation related to ${\hat{D}}_{C}$, and $\underset{C}{⋃}pool_{C}\left(conv_{C}\left({\hat{D}}_{C}\right)\right)$ represents the merging of all feature maps that have undergone pooling and convolution operations. When using the additive fusion method, the pooling type is max pooling, the convolution kernel size is 3 × 3, the stride value is 1, and the activation function is ReLU. To further enhance the effectiveness of feature fusion, ResNet50 has also been improved by introducing the Switch module and referencing the idea of feature pyramid. Therefore, the improved structure of ResNet50 is shown in Fig 4.

From Fig 4, the improved ResNet50 structure includes different feature image inputs, convolutional layers, max pooling layers, Switch modules, four residual modules, average pooling layers, and output layers. In the four residual modules, each module contains several convolutional layers and skip connections. The core of this design is to directly add input to output through skip connections, effectively solving the problem of gradient vanishing in deep network training, enabling the network to learn residual mapping, and improving the efficiency and effectiveness of feature extraction. The Switch module draws on the idea of feature pyramids to adjust the number and size of channels in feature maps, thereby achieving the fusion of features at different levels. The Switch module is a custom module that performs channel unification, size matching, and effective feature filtering on multi-scale feature maps output by different residual modules of ResNet50. Its architecture is “feature input layer → 1 × 1 convolutional channel adjustment layer (ReLU activation) → bilinear interpolation size adaptation layer → Sigmoid attention weighting layer → feature output layer”. At the same time, this module refers to the core idea of “multi-scale feature fusion” in the feature pyramid, extracts different scale features through different convolution modules of ResNet50, and then standardizes and fuses cross scale features through this module, similar to the “top-down+horizontal connection” mechanism of the feature pyramid, allowing the fused features to have both global positioning and local detail resolution capabilities. Although these fused deep features contain rich information of the image, they also bring high-dimensional problems. High dimensional features not only increase the time and computational resources required for model training, but also lead to overfitting of the model. Therefore, to tackle the challenge of high-dimensional features, the study adopted a regularized self representation unsupervised feature selection method. The fundamental concept of this approach is to select the most representative features through regularization constraints, thereby achieving dimensionality reduction of features. It has the advantages of preserving the inherent structure of data, strong scalability, and high flexibility [27,28]. In this method, each feature $F_{G}$ in the high-dimensional dataset E can be expressed as a linear combination, as shown in equation (9).


$$F_{G}=\sum_{H=1}^{I}F_{G}J_{HG}+K_{G}$$ (9)

In equation (9), G represents the G th column of the dataset matrix E, and I represents the number of features, which is the total number of columns in the matrix. $J_{HG}$ represents the weight coefficient, and $K_{G}$ represents the error term. H is an index variable for summing symbols, mainly used to traverse all features in the dataset. When constructing a high-dimensional feature matrix E, it is necessary to first obtain the ResNet50 output features, and then arrange the ResNet50 output feature vectors of all samples in the feature library in columns to form a high-dimensional feature matrix. Next, perform column normalization on the high-dimensional feature matrix E. In addition, the position of the regularized self representation feature selection step in the overall process is after ResNet50 feature extraction and feature matrix E construction, and before the input of the Elman network, that is, the reduced dimensional feature matrix is the input of the Elman network. Meanwhile, the expression of E is shown in equation (10).


E=ER+M (10)

In equation (10), R represents the coefficient matrix and M represents the residual error matrix. To remove the trivial solution of R, regularization term N(R) was used in the study. Therefore, the adjustedexpression $R^{\prime }$ of R is shown in equation (11).


$$R^{\prime }=\mathrm{\backslash arg}\underset{r}{\min}{\left\|E-ER\right\|}_{2,1}+ON\left(R\right)$$ (11)

In equation (11), $\mathrm{\backslash arg}\underset{r}{\min}$ represents finding r that minimizes the objective function, r is the data in R, and ${\left\|E-ER\right\|}_{2,1}$ is the $L_{2,1}$ norm of the E−ER matrix, which is used to measure the error of the feature self representation model. O represents the regularization parameter. To solve $R^{\prime }$, the iterative reweighted least squares algorithm was used in the study. This algorithm is an iterative method used to optimize nonlinear problems, which has the advantages of reducing the influence of outliers, handling heteroscedasticity, flexibly adapting to different loss functions, fast convergence, and high computational efficiency [29]. After updating $R^{\prime }$, feature ranking is performed using feature weights, and the image feature library is established by selecting the preceding features. Finally, in the image feature library, each position coordinate has corresponding depth feature data, which provides data support for the subsequent construction of indoor position perception models.

### 3.3. Construction of indoor position perception model considering Elman parameter optimization

**Fig 5:**
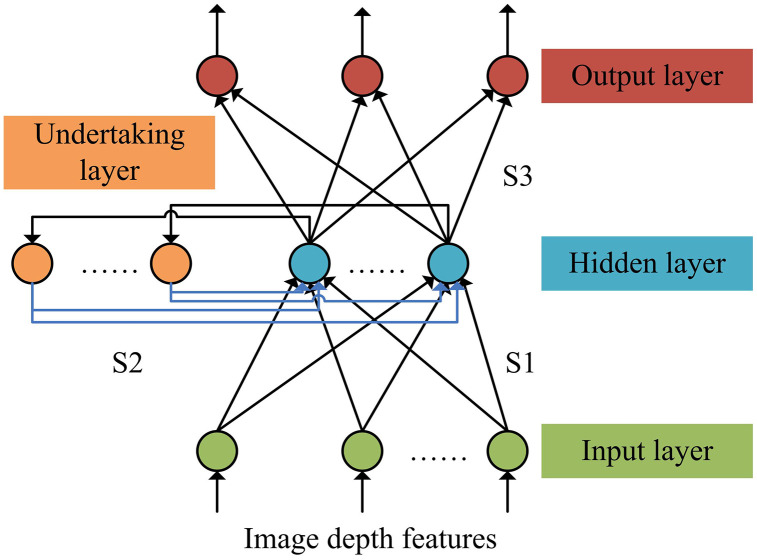
The Elman indoor position perception model.

Before carrying out the construction of the indoor location perception model, the research already completed the design of data collection methods and feature extraction based on Resnet50. The completion of these two basic tasks provides indispensable data support and feature representation for the construction of indoor position perception models. Therefore, based on this, the strategy adopted in the study is to organically combine the extracted deep features with Elman neural networks(NN). This design decision is based on a deep understanding of task requirements and a meticulous consideration of model performance. Common methods for constructing indoor location perception models include Kalman filtering, particle filtering, support vector machines, naive Bayes, K-nearest neighbors, and long short-term memory networks. However, these methods all have certain shortcomings, such as Kalman filtering being suitable for linear systems, particle filtering being suitable for nonlinear non Gaussian systems, and support vector machines being good at small sample classification. In contrast, Elman neural networks have dynamic memory capabilities and can effectively process time series data, making them suitable for indoor positioning with dynamic signal changes. In addition, to further improve the performance of Elman NN, the research adopted the IGWO algorithm for parameter optimization. Elman NN is a feedforward NN with dynamic memory function, which can model and predict time series data, and is suitable for indoor position perception tasks that require consideration of time series information [30,31]. In addition, due to the presence of a receptive layer, Elman NNs can alleviate problems such as gradient vanishing or exploding. Therefore, based on the deep features extracted by Resnet50, an Elman indoor position perception model was constructed, as shown in Fig 5.

From Fig 5, the model includes an input layer, a hidden layer, a receiver layer, and an output layer. Among them, the input data of the input layer is the image depth features obtained by Resnet50, while the hidden layer performs nonlinear transformation on the input features, and the output layer needs to output the position coordinates. In addition, S1, S2, and S3 all represent connection weights, and the activation function of the hidden layer is the Sigmoid function. After building the model, it is necessary to train the Elman NN. This process is achieved by adjusting the weights and thresholds of the network to minimize the error between the network’s output and the true position coordinates. However, the initial weights and threshold parameters have a significant impact on the performance of Elman NNs. Firstly, the initial weights determine the learning direction and speed of the network at the beginning of training. Inappropriate initial weights may lead to slow convergence speed of the network, or even inability to converge. Secondly, the initial weights and thresholds can affect the stability of the network. If the initial value is set improperly, the network may experience oscillations or divergence during the training process. In addition, the initial weights and thresholds also determine whether the network can effectively find the global optimal solution. Traditional Elman NNs typically use random initialization methods, which can lead to the network easily falling into local optima during training, especially when dealing with complex problems, which is more common. To overcome this problem, an improved GWO algorithm was designed to optimize the initial weights and thresholds of Elman NNs. The GWO algorithm is a nature-inspired optimization technique that mimics the predatory behavior of gray wolf packs, featuring a straightforward principle, uncomplicated implementation, few parameters that need to be adjusted, and a strong global search ability. It finds extensive utilization in practical problems such as workshop scheduling, parameter optimization, image classification, and path planning [32]. The mathematical model of GWO algorithm is shown in equation (12).


$$\left\{ \begin{array}{c} \vec{T}=\left|\vec{U}{\vec{V}}_{W}(k)-\vec{V}(k)\right|\\ \vec{V}(k+1)={\vec{V}}_{W}(k)-\vec{j}\vec{T} \end{array}\right.$$ (12)

In equation (12), $\vec{T}$ represents the distance between grey wolf prey, $\vec{U}$ is a random vector, and there exists $\vec{U}=2\times {\vec{t}}_{1}$. ${\vec{t}}_{1}$ represents a random number in the [0,1] interval. ${\vec{V}}_{W}(k)$ represents the position vector of the prey, and $\vec{V}(k)$ represents the position vector of the gray wolf. k represents the current number of iterations, and $\vec{j}$ represents the key control parameters. Meanwhile, in the GWO algorithm, the position updates of the top three optimal solutions are shown in equation (13).


$$\left\{ \begin{array}{c} {\vec{T}}_{\alpha }=\left|{\vec{U}}_{1}{\vec{V}}_{\alpha }-\vec{V}\right|\\ {\vec{T}}_{\beta }=\left|{\vec{U}}_{2}{\vec{V}}_{\beta }-\vec{V}\right|\\ {\vec{T}}_{\delta }=\left|{\vec{U}}_{3}{\vec{V}}_{\delta }-\vec{V}\right| \end{array}\right.$$ (13)

In equation (13), α, β, and δ represent the first three optimal solutions, respectively, and ${\vec{U}}_{1}$, ${\vec{U}}_{2}$, and ${\vec{U}}_{3}$ are all random vectors. ${\vec{T}}_{\alpha }$, ${\vec{T}}_{\beta }$, and ${\vec{T}}_{\delta }$ represent the distances between the current positions of α, β, and δ respectively. ${\vec{V}}_{\alpha }$, ${\vec{V}}_{\beta }$, and ${\vec{V}}_{\delta }$ represent the current position vectors of α, β, and δ, respectively. However, the GWO algorithm also has some shortcomings, such as difficulty in escaping local optima and possibly slow convergence speed. To overcome these shortcomings, research mainly improves it from three aspects: chaos initialization, improving convergence factor μ, and chaos disturbance strategy. Chaos initialization can increase the diversity of individual populations, improve the global search ability of algorithms, avoid algorithms falling into local optima, and enhance overall convergence speed. Therefore, the study adopted Tent mapping for chaos initialization. The traversal of Tent mapping has uniformity and randomness, which can make it easier for the algorithm to escape from local optima and improve its global search capability. The expression of Tent mapping is shown in equation (14).


$$\phi (k+1)=\left\{ \begin{array}{cc} 2\phi (k), & 0⩽\phi (k)⩽0.5\\ 2(1-\phi (k)), & 0.5⩽\phi (k)⩽1 \end{array}\right.$$ (14)

In equation (14), ϕ(k) represents the variable value at the k th iteration. Improving the convergence factor μ is to change the initial linear μ to nonlinear μ, and the study uses a sine function. Therefore, the expression of $\mu^{\prime }$ after improvement is shown in equation (15).


$$\mu^{\prime }=2-2\sin{\left(\frac{\pi }{\text{2}}\times \frac{k}{k_{\max}}\right)}^{2}$$ (15)

In equation (15), $k_{\max}$ represents the maximum number of iterations. After improvement, $\mu^{\prime }$ achieves dynamic adjustment through sine function variation, aiming to achieve a better balance between global and local search, thereby enhancing the accuracy of local search while maintaining global search capability, and improving the overall performance of the algorithm. Chaos disturbance strategy is a method of introducing chaos mechanisms into optimization algorithms or control systems to enhance search capabilities, avoid local optima, or improve system dynamic characteristics. Therefore, studying the chaotic sequence $\theta_{tent}$ obtained through Tent mapping to add chaotic disturbances in α, β, and δ. The expression of this strategy is shown in equation (16).


$$\theta^{\prime }=\gamma \theta_{tent}+\left(1-\gamma \right)\theta$$ (16)

In equation (16), θ is the position of the individual with added disturbance, and denotes the position $\theta^{\prime }$ of the individual with added disturbance. γ is the perturbation factor, expressed as equation (17).


$$\gamma =\mathrm{lg}\left(\frac{k+9}{k}\right)$$ (17)

**Fig 6:**
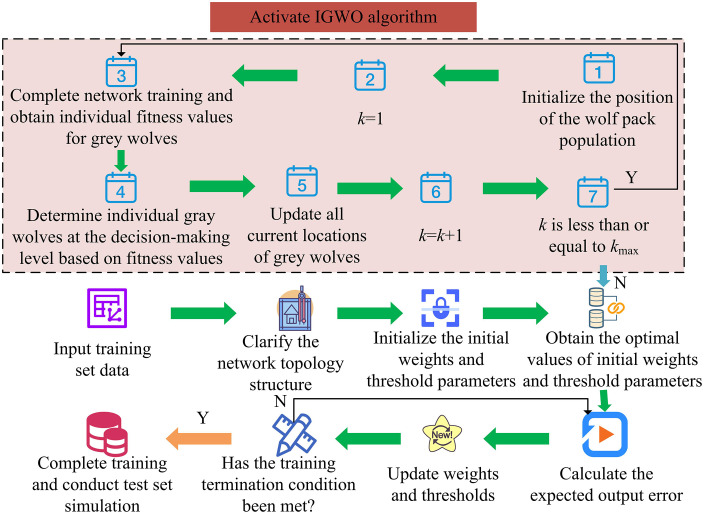
Flowchart of IGWO-ElmanNN.

The innovation of IGWO lies in the collaborative optimization of improvement strategies, rather than the innovation of a single improvement point. For example, the coordination of chaos initialization and chaos disturbance: the traditional chaos improvement is only used for initialization or disturbance. IGWO combines the Tent map chaos initialization and chaos disturbance to form the whole process chaos optimization of “initialization iteration disturbance”. Chaos initialization avoids initial population concentration, non-linear μ balance global/local search in high-dimensional space, and chaos disturbance breaks through local optima in high-dimensional space. The synergy of the three makes IGWO perform better than traditional improved GWO in high-dimensional optimization. The study aims to optimize the initial weights and thresholds of Elman NNs through IGWO, with the aim ofenhancing the training capability of Elman NNs. The flowchart of IGWO-ElmanNN is presented in Fig 6.

**Fig 7:**
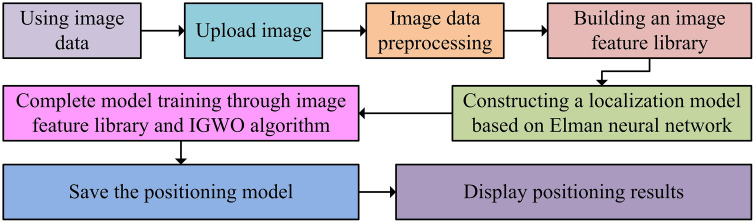
The overall construction process of indoor position perception model.

From Fig 6, the key step in the IGWO-ElmanNN process is to start the IGWO algorithm to obtain the optimal values of initial weights and threshold parameters, and input them into the Elman NN. Through this step, the influence of randomly initialized initial weights and threshold parameters on Elman NNs can be overcome. The overall construction process of the indoor position perception model is shown in Fig 7.

From Fig 7, the key steps in the overall construction of the indoor position perception model are collecting image data, constructing an image feature library, and constructing a localization model based on Elman NN. Among them, data collection provides high-quality image data for the subsequent steps, the feature library is constructed to establish a “feature position” mapping relationship, and the positioning model achieves position prediction by learning this mapping. The three links are sequentially connected and mutually supported, forming a complete perception link from physical signals to positional information. Improving the feature extraction ability of ResNet50 and optimizing the parameters of Elman by IGWO are the core supports for improving positioning accuracy and stability. Through this model, the mapping relationship between position and image features can be accurately learned from the feature library, thereby accurately predicting the corresponding position coordinates in practical applications and achieving high-precision indoor positioning.

## 4. Results

To confirm the capability of the designed feature extraction method and visible light indoor position perception model, the experiment environment, setup, and dataset were described, and the comparison methods for each method were selected. In addition, the study also set the parameters of the algorithm.

### 4.1. Algorithm performance validation

**Fig 8:**
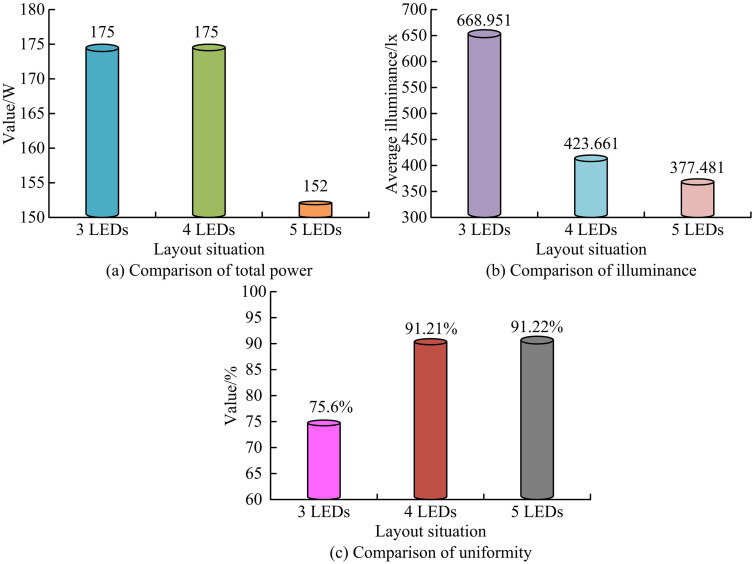
Comparison results of different light source layout methods.

To analyze the rationality of selecting light source layouts, the study set up three common layouts and analyzed their corresponding total power and uniformity. In addition, in terms of the accuracy of feature extraction using Resnet50, the study used cosine similarity to calculate the similarity between two feature vectors and considered the time required for feature extraction. The study selected Inception v3, Visual Geometry Group 16 layer network (VGG-16), Residual Network-18 (ResNet-18), and MobileNet-V1 for comparison. Meanwhile, the Resnet50 performance validation used a dataset of image data collected for research, with 2000 images preprocessed. The original resolution of the visible light image is 1280 × 720 pixels, with a pixel size of 1.4μm × 1.4μm and an interpolation scaling factor of 0.175. The value of the regularization parameter O is 0.01, the iteration stopping criterion is a loss function change < 1e-5, and the feature selection threshold is 0.8. Subsequently, in the performance verification of the IGWO algorithm, the CEC2013 test function set was used in the study [33], which includes single peak test function f2, multi-peak test functionf9, and composite functionf24.The test functions f2, f9, and f24 were standard functions in the CEC2013 test function set, which are widely used for performance evaluation of optimization algorithms due to their unique characteristics. F2, as a unimodal function, was mainly used to test the local and global search capabilities of algorithms. F9, as a multimodal function, was used to evaluate the algorithm’s global optimization ability and ability to avoid local optima. As a composite function, f24 was used to test the comprehensive performance of the algorithm in complex environments. The selection of these functions in this study to validate the performance of the IGWO algorithm was based on their representativeness and universality in optimizing algorithm evaluation. In optimization algorithms, fitness values were used to measure the performance of the algorithm on specific solutions, while standard deviation reflects the stability and consistency of the algorithm’s results after multiple runs. In the testing function, finding the minimum value of fitness value and standard deviation was to find the optimal or near optimal solution and ensure consistent performance of the algorithm in different runs. This was consistent with the goal of optimization algorithms, which was to find the parameter combination that minimizes the value of the objective function. Therefore, the research needed to find the minimum values of fitness and standard deviation on the test function to evaluate algorithm performance. The Elman neural network has a 4-layer structure, with 614 neurons in the input layer, 15 neurons in the hidden layer, 15 neurons in the receiving layer, and 3 neurons in the output layer, with a time step of 5. In addition, the Sigmoid activation function adopts the standard Logistic Sigmoid function. The parameter initialization range of the IGWO algorithm is [−1, 1], and the population size is 60. The two random vectors in this algorithm are used to control the search step size and adjust the direction of gray wolf position updates. Among them, the random vector used to control the search step size is a multidimensional vector with element values ranging from [0,1], and its dimension is consistent with the parameters to be optimized. The random vector used to adjust the update direction of the gray wolf position is a multidimensional vector with element values ranging from [0,2], and is randomly generated at each iteration. In addition, for the comparison algorithm of IGWO algorithm, the study selected Particle Swarm Optimization (PSO), Genetic Algorithm (GA), GWO algorithm, and a combination algorithm 1 that combines random black hole strategy and GWO.Meanwhile, the initial population size of IGWO and the comparison algorithm were both 60, and the maxiteration countwas 1200.In the experimental environment, the study used the Windows 10 operating system, with an Intel Core i5-13500 central processor, a maximum turbo frequency of 4.8GHz, 20 threads, and a maximum memory bandwidth of 76.8GB/s. In the feature extraction comparison experiment, the training schemes for all benchmark models (Inception v3, VGG-16, ResNet-18, MobileNet-V1) were unified as follows: the optimization algorithm used Adam optimizer, the initial learning rate was 0.001, the training epochs were 5, and the batch size was 32. The comparison results of different light source layout methods are shown in Fig 8.

From Fig 8 (a), the horizontal axis represents the layout of different numbers of LEDs, and the vertical axis represents their respective total power. In terms of total power comparison, the values corresponding to 3, 4, and 5 LED layouts were 175W, 175W, and 152W, respectively. The phenomenon of an increase in the number of LEDs but a decrease in total power was due to the use of more efficient LEDs, which reduced the power demand of individual LEDs. The total power of 3 and 4 LEDs was the same, because in both layouts, the power of each LED was adjusted to meet the same total power demand. From Fig 8 (b), as the number of LEDs increased, the corresponding illuminance decreased. This might be because although the number of LEDs increased, the total power was limited and remained unchanged, resulting in a decrease in the power allocated to each LED, thereby reducing the brightness output of each LED. From Fig 8 (c), as the number of LEDs increased, the uniformity of illumination significantly improved, indicating a more balanced distribution of illumination. In addition, the uniformity values of the 4 and 5 LED layouts were relatively close. Therefore, taking into account the above factors and cost considerations, the study selected four LED layouts.The performance verification results of the research design feature extraction method are presented in Table 2.

**Table 2:** Performance verification results of feature extraction methods.

Method	Cosine similarity					Time consuming/ms					Reference
	Quantity of trials					Quantity of trials					
	1	2	3	4	5	1	2	3	4	5	
Inception v3	0.853	0.836	0.871	0.811	0.855	107	113	111	100	106	[34]
VGG-16	0.782	0.776	0.753	0.869	0.825	104	112	90	96	82	[35]
ResNet-18	0.762	0.736	0.812	0.713	0.847	88	84	82	86	86	[36]
MobileNet-V1	0.669	0.658	0.648	0.676	0.632	55	64	74	55	59	[37]
Based on Resnet50	0.059	0.135	0.119	0.017	0.186	46	46	30	53	40	This study

From Table 2, the average cosine similarity valuecorrespondingto the Resnet50 extraction method in the research design was 0.103, while the average cosine similarity valuescorresponding to Inception v3, VGG-16, ResNet-18, and MobileNet-V1were 0.845, 0.801, 0.774, and 0.657, respectively. The range of cosine similarity values is [−1, 1], where 1 indicates that the feature vectors are completely identical, 0 indicates no correlation, and −1 indicates opposite directions. Feature library diversity refers to the significant differences in feature vectors at different locations, which should be as uncorrelated as possible, i.e., close to 0, otherwise it is prone to localization errors. The cosine similarity of the research design feature extraction method was closer to 0, indicating that the feature library constructed by this method was more diverse, avoiding feature duplication and determining the uniqueness of features. This might be because the residual structure of Resnet50 was capable of extracting deep features and combines the Switch module to achieve feature fusion, thereby enhancing the performance of research and design feature extraction methods. The computational complexity and resource requirements of different feature extraction methods are compared in Table 3.

**Table 3:** Comparison of computational complexity and resource requirements of different feature extraction methods.

Method	Floating-point Operations Per second (FLOPs)/×10^9^				Memory usage/MB				Reference
	Number of experiments				Number of experiments				
	1	2	3	4	1	2	3	4	
Inception v3	5.5	5.8	5.7	5.6	189	193	192	191	[34]
VGG-16	15.2	15.6	15.5	15.4	253	258	256	254	[35]
ResNet-18	1.7	1.8	1.8	1.7	94	97	96	95	[36]
MobileNet-V1	0.54	0.56	0.57	0.55	62	64	65	63	[37]
Based on Resnet50	3.9	4.2	4.1	4.0	125	129	128	126	This study

**Fig 9:**
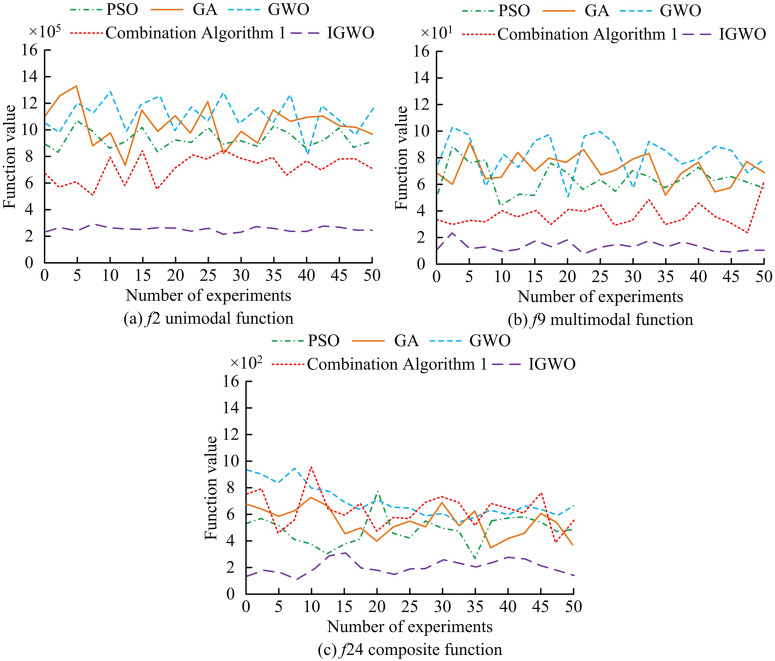
Comparison of fitness values of various algorithms on different test functions.

From Table 3, it can be seen that the range of floating-point operations based on ResNet50 method is 3.9 × 10^9^–4.2 × 10^9^, and the range of memory usage is 125MB-129MB. It can be seen that the research design based on Resnet50 extraction method has achieved a good balance between computational complexity and memory usage. Compared with Inception-v3 and VGG-16, it has lower computational overhead. Compared with ResNet-18 and MobileNet-V1, it can extract richer features with better performance and is more suitable for practical application needs. The comparison of fitness values of various algorithms on different test functions is shown in Fig 9.

From Fig 9 (a),on the unimodal test functionf2, The maximum fitnessvalue of the IGWO algorithm was 2.97 × 10^5^, and the average fitness valuesofPSO, GA, GWO, and combination algorithm 1were 9.52 × 10^5^, 10.73 × 10^5^, 11.26 × 10^5^, and 7.69 × 10^5^. As presented in Fig 9 (b), on the multi-modal test functionf9, the average fitness valuesof the five algorithms were 1.47 × 10^1^, 6.32 × 10^1^, 7.55 × 10^1^, 7.98 × 10^1^, and 3.91 × 10^1^, respectively. From Fig 9 (c), on the composite function f24, the average fitness value was larger. The GWO algorithm had a fitness value of 6.94 × 10^2^, while the IGWO algorithm had a smaller fitness value of 2.17 × 10^2^. Overall, under different test functions, the fitness value of the IGWO algorithm was notably lower than that of the comparison algorithm. This indicated that the IGWO algorithm could more effectively explore the search space and find points closer to the global optimal solution. This might be due to the introduction of chaos initialization and hybrid disturbance strategies in the IGWO algorithm, combined with improved convergence factors, to enhance population diversity and balance global local search, thereby improving the algorithm’s optimization ability under different test functions. The standard deviation comparison of various algorithms on different test functions is shown in Table 4.

**Table 4:** Comparison of standard deviations of various algorithms on different test functions.

Algorithm	Unimodal function f2						
	Quantity of trials						
	1	2	3	4	5	6	7
PSO	8.12 × 10^3^	8.06 × 10^3^	8.33 × 10^3^	8.05 × 10^3^	8.26 × 10^3^	8.18 × 10^3^	8.04 × 10^3^
GA	8.63 × 10^3^	8.57 × 10^3^	8.91 × 10^3^	8.86 × 10^3^	8.75 × 10^3^	8.71 × 10^3^	8.73 × 10^3^
GWO	9.28 × 10^3^	9.66 × 10^3^	9.43 × 10^3^	9.75 × 10^3^	9.26 × 10^3^	9.98 × 10^3^	9.54 × 10^3^
Combination algorithm 1	7.96 × 10^3^	7.16 × 10^3^	8.01 × 10^3^	7.64 × 10^3^	7.72 × 10^3^	7.45 × 10^3^	7.27 × 10^3^
IGWO (manuscript)	6.33 × 10^3^	6.48 × 10^3^	6.17 × 10^3^	6.09 × 10^3^	6.25 × 10^3^	6.49 × 10^3^	6.35 × 10^3^
Algorithm	Multi peak function f9						
	Quantity of trials						
	1	2	3	4	5	6	7
PSO	7.95 × 10^0^	8.49 × 10^0^	8.31 × 10^0^	8.07 × 10^0^	8.22 × 10^0^	8.11 × 10^0^	8.42 × 10^0^
GA	8.98 × 10^0^	9.02 × 10^0^	8.65 × 10^0^	8.60 × 10^0^	8.77 × 10^0^	8.80 × 10^0^	9.15 × 10^0^
GWO	9.62 × 10^0^	9.80 × 10^0^	9.99 × 10^0^	9.75 × 10^0^	9.81 × 10^0^	9.94 × 10^0^	9.88 × 10^0^
Combination algorithm 1	6.98 × 10^0^	7.13 × 10^0^	7.25 × 10^0^	7.34 × 10^0^	7.09 × 10^0^	7.41 × 10^0^	7.25 × 10^0^
IGWO (manuscript)	6.19 × 10^0^	5.95 × 10^0^	5.48 × 10^0^	6.33 × 10^0^	5.64 × 10^0^	6.19 × 10^0^	6.20 × 10^0^
Algorithm	Mixed function f24						
	Quantity of trials						
	1	2	3	4	5	6	7
PSO	6.78 × 10^1^	6.09 × 10^1^	6.33 × 10^1^	6.59 × 10^1^	6.72 × 10^1^	6.94 × 10^1^	6.86 × 10^1^
GA	7.52 × 10^1^	7.25 × 10^1^	7.63 × 10^1^	7.88 × 10^1^	7.39 × 10^1^	7.58 × 10^1^	7.61 × 10^1^
GWO	8.66 × 10^1^	8.17 × 10^1^	8.35 × 10^1^	8.48 × 10^1^	8.65 × 10^1^	8.90 × 10^1^	8.63 × 10^1^
Combination algorithm 1	5.55 × 10^1^	5.71 × 10^1^	5.63 × 10^1^	5.22 × 10^1^	5.49 × 10^1^	5.78 × 10^1^	5.36 × 10^1^
IGWO (manuscript)	4.82 × 10^1^	4.95 × 10^1^	5.11 × 10^1^	4.34 × 10^1^	4.81 × 10^1^	4.95 × 10^1^	4.66 × 10^1^

From Table 4, on the unimodaltest functionf2, the minimumstandard deviationof the IGWO algorithmwas 6.09 × 10^3^, and the average standard deviation was 6.31 × 10^3^. Meanwhile, the average standard deviations of PSO, GA, GWO, and combinationalgorithm1were 8.15 × 10^3^, 8.74 × 10^3^, 9.56 × 10^3^, and 7.60 × 10^3^, respectively, all greaterthan 6.31 × 10^3^. In addition, on the multi-modal test functionf9 and the compositefunctionf24, the standard deviation of the IGWO algorithm was notably smaller than that of the comparison algorithm, with values ranging from [5.48 × 10^0^, 6.33 × 10^0^] and [4.34 × 10^0^, 5.11 × 10^0^], respectively. In summary, the standard deviations of the IGWO algorithm under different test functions were also notably smaller. The deviation was notably smaller than the comparison algorithm, and the fluctuation rangewas smaller. This indicated that the IGWO algorithm could achieve consistent optimization results in multiple runs and maintain stable performance under different test functions, without significant performance fluctuations due to the complexity or randomness of the functions. This also indicated that the algorithm could stably find near optimal solutions in various complex optimization scenarios, and was not easily affected by local optimal solutions. The ablation experimental results of the IGWO algorithm are shown in Table 5.

**Table 5:** The ablation experimental results of the IGWO algorithm.

GWO	Chaos initialization	Improve convergence factor μ	Chaos disturbance strategy	Minimum standard deviation		
*f2*	*f9*	*f24*
√	√	√	√	6.11 × 10^3^	6.08 × 10^0^	4.85 × 10^1^
√	√	√	×	6.98 × 10^3^	7.14 × 10^0^	5.39 × 10^1^
√	√	×	×	7.24 × 10^3^	7.86 × 10^0^	5.98 × 10^1^
√	×	×	×	7.98 × 10^3^	8.35 × 10^0^	6.13 × 10^1^

From Table 5, it can be seen that in the ablation experiment of the IGWO algorithm, when chaos initialization, improved convergence factor μ, and chaos disturbance strategy are used simultaneously, the minimum standard deviations of the algorithm under different test functions *f*2, *f*9, and *f*24 are 6.11 × 10^3^, 6.08 × 10^0^, and 4.85 × 10^1^, respectively. If there is a lack of chaos disturbance strategy, the minimum standard deviation of each function increases; If there is a lack of improved convergence factor μ, the minimum standard deviation of each function will further increase; If all three improvements are lacking, the minimum standard deviation of each function will be the largest. It can be seen that under the synergy of the three improvements, the performance of the IGWO algorithm is better.

The comparative experiments of different light source layout methods in the algorithm performance verification section verified the superiority of the selected layout scheme in terms of uniformity and lighting effect, providing a good foundation for the positioning system. Moreover, by conducting a comparative analysis of various feature extraction methods and rigorously testing optimization algorithms, the merits of the proposed approach in constructing feature libraries and optimizing parameters were clearly demonstrated. This directly led to significant enhancements in both the accuracy and stability of indoor positioning.

### 4.2. Analysis of example results

**Fig 10:**
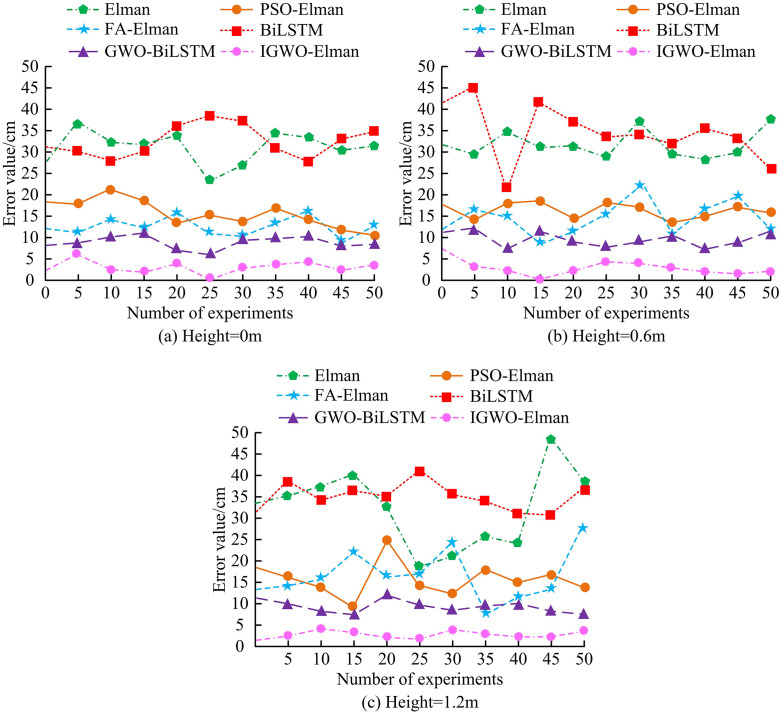
The static analysis results of various algorithms.

To verify the performance of the IGWO-ElmanNN indoor position perception model designed for research, four LEDs were arranged on a 4m × 4m × 3m indoor model, and different heights (0m, 0.6m, and 1.2m) of image data were collected through telescopic rods. The sampling point interval was 0.1m, and the LED power was 8W. In terms of simulation parameters, the wall reflection coefficient is 0.7, the ground reflection coefficient is 0.5, and the ceiling reflection coefficient is 0.8. The illuminance calculation grid is 0.1m × 0.1m for indoor ground, with a total of 1600 points. In the quantitative design standards for optimizing light source layout, the study mainly referred to the illumination uniformity and total power efficiency of different layouts. The values of these parameters are based on empirical research [38]. In addition, the study used a Logitech C930e model camera and an Intel Core i5-13500 central processor. The training and testing datasets are from two non overlapping physical environments, with the training dataset consisting of 2000 visible light images collected in the laboratory environment on the 1st and 2nd floors of the experimental building. The test dataset consists of 800 visible light images, collected in the actual application area on the third floor of the experimental building. The experiment mainly conducted three aspects of testing, namely static analysis, dynamic analysis, and environmental light impact analysis. Among them, in static analysis, the study focused on single point acquisition of image data through cameras. In terms of dynamic analysis, the study utilized the Logitech C920 HD Pro image sensor and the Makeblock mBot Ranger motion car, and designed a 2.0m × 2.0m motion trajectory. In the analysis of the impact of ambient light, the study simulated testing environments with and without external light, and selected 10 points for comparison. Under conditions without external light, all external light sources were turned off, and illumination was provided solely by four LED light sources. The TES-1332A lux meter was used to measure light intensity at each collection point, resulting in an average illuminance of 250 lux. Under conditions with external light, a scenario combining natural light and indoor lighting was simulated by opening windows and turning on two 40W fluorescent lamps, yielding an average illuminance of 800 lux at each collection point. Meanwhile, after collecting image data, the study conducted preprocessing on it. To further validate the performance of the IGWO Elman NN indoor position perception model, the study selected a multi story building from a certain university and localized it in three aspects. In addition, the learning rate of IGWO-ElmanNN was 0.01 and the size of the hidden layer was 15. When training the IGWO Elman NN indoor position perception model, the Elman network weights and thresholds were randomly initialized first, and then the IGWO algorithm was used for optimization to determine the optimal initial value. During training, using image feature library data as input and corresponding position coordinates as output, network parameters were adjusted to minimize prediction errors. In the comparison method of IGWO-ElmanNN, Elman, PSO-Elman, Firefly Algorithm (FA)-Elman, Bidirectional Long Short-Term Memory (BiLSTM) NN, and GWO-BiLSTM were selected for the study. In terms of model details, Elman has a 614 dimensional input layer, 15 neurons in the hidden layer, and 15 neurons in the receptive layer. The PSO population size in PSO Elman is 60, and the inertia weight is 0.7. Meanwhile, the population size of FA in FA Elman is 60, with an attractiveness of 0.5 and a light absorption coefficient of 0.2. All comparison models were trained and tested on the same dataset as IGWO Elman, with only different model architectures to ensure fairness in comparison, and the testing equipment and data preprocessing methods were completely consistent. In addition, to verify performance differences, the study used independent sample *t*-test. The static analysis results of various algorithms are shown in Fig 10.

From Fig 10 (a), when the height was 0m, the maximum error of the IGWO-ElmanNN was 6.12 cm. In addition, the average errors of Elman, PSO-Elman, FA-Elman, BiLSTM NN, and GWO-BiLSTMNN were 32.52 cm, 15.65 cm, 14.19 cm, 33.98 cm, and 9.17 cm, respectively. From Fig 10 (b), when the height was 0.6m, the IGWO-ElmanNN had a smaller error with an average of 3.57 cm, while the BiLSTMNN had a larger error with an average of 34.85 cm. As shown in Fig 10 (c), when the heightwas 1.2m, The average errors of the IGWO-ElmanNN and five comparison methods were 3.19 cm, 33.75 cm, 16.01 cm, 15.42 cm, 35.18 cm, and 9.63 cm, respectively. In summary, at different heights, the errors of the IGWO-ElmanNN were notably lower than those of the comparison methods. This might be because IGWO enhanced the initial weights and thresholds of the Elman NN through chaos initialization, thereby improving convergence factors and employing chaos disturbance strategies. These enhancements effectively avoided the issue of the Elman NN tending to fall into local optima during training. Consequently, the model’s global search capability and convergence speed were improved, allowing the IGWO-Elman NN to more accurately perceive indoor positions at varying heights. In addition, all error differences have a significance of *p* < 0.05, indicating that these differences are statistically significant and that the performance advantage of IGWO Elman neural network is not caused by random factors. The error distribution of various algorithms is shown in Table 6.

**Table 6:** Error distribution of various algorithms.

Algorithm	Height = 0m											
	Distribution of positioning errors/cm											
	0	5	10	15	20	25	30	35	40	45	50	55
Elman	1	3	2	7	11	117	120	123	80	0	0	2
PSO-Elman	3	45	98	115	17	13	9	14	13	4	1	0
FA-Elman	2	33	75	113	12	11	15	8	4	3	0	0
BiLSTM	0	4	8	21	52	68	78	105	19	3	1	0
\	Distribution of positioning errors/cm											
\	0	1	2	3	4	5	6	7	8	9	10	11
GWO-BiLSTM	1	2	1	3	6	7	2	8	12	23	19	15
IGWO-Elman	10	15	8	14	9	3	3	2	1	0	0	0
Algorithm	Height = 0.6m											
	Distribution of positioning errors/cm											
	0	5	10	15	20	25	30	35	40	45	50	55
Elman	9	1	7	2	62	117	120	122	45	17	1	3
PSO-Elman	5	60	95	107	40	37	8	4	3	0	1	0
FA-Elman	12	33	62	105	53	11	4	0	0	1	0	1
BiLSTM	9	1	4	9	12	45	68	106	55	12	1	0
\	Distribution of positioning errors/cm											
\	0	1	2	3	4	5	6	7	8	9	10	11
GWO-BiLSTM	3	1	0	0	4	1	7	12	18	27	25	17
IGWO-Elman	18	11	17	25	13	2	1	1	0	0	0	0
Algorithm	Height = 1.2m											
	Distribution of positioning errors/cm											
	0	5	10	15	20	25	30	35	40	45	50	55
Elman	7	0	4	1	3	47	95	127	110	40	0	1
PSO-Elman	7	45	88	112	37	16	4	4	2	1	0	0
FA-Elman	12	23	76	103	46	9	7	1	1	0	2	1
BiLSTM	17	3	1	0	11	32	48	107	60	12	0	0
\	Distribution of positioning errors/cm											
\	0	1	2	3	4	5	6	7	8	9	10	11
GWO-BiLSTM	8	3	0	0	1	1	8	11	22	30	23	21
IGWO-Elman	20	9	23	21	19	4	2	0	0	0	0	0

**Fig 11:**
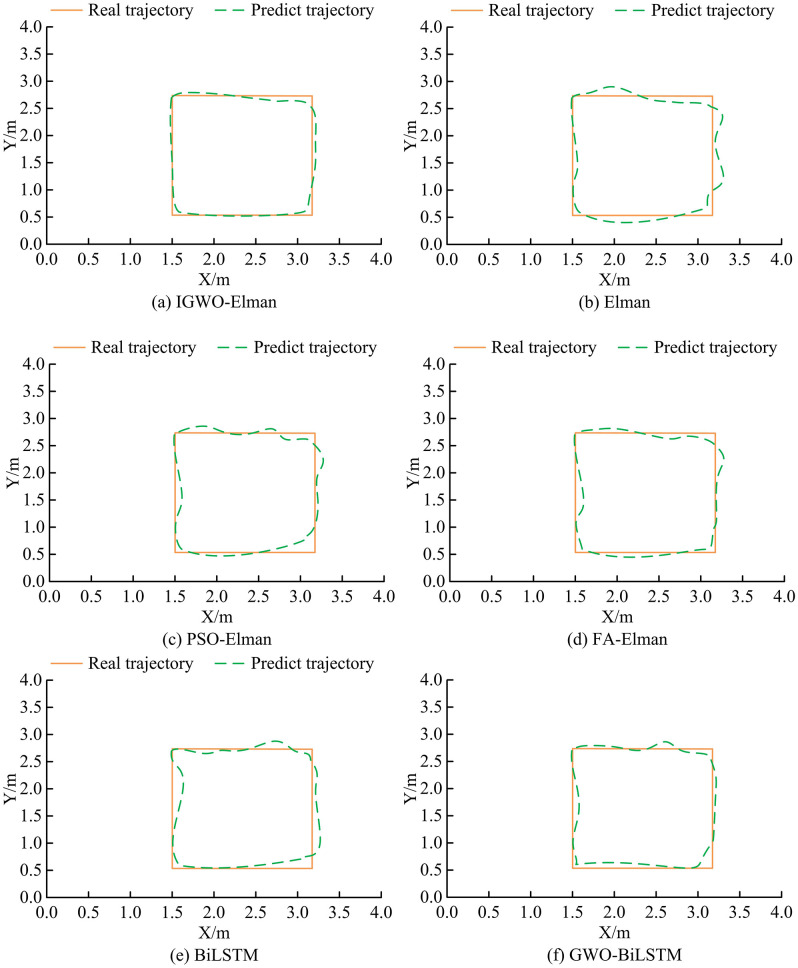
The dynamic analysis results of different methods.

From Table 6, when the height was 0m, the error of IGWO-ElmanNN was mainly distributed within the range of (0 cm, 4 cm).Meanwhile, the most frequent error value of this method was 1 cm, and the specific frequency was 15 times. In addition, the error distributions of Elman, PSO-Elman, FA-Elman, BiLSTM NN, and GWO-BiLSTM were (20 cm, 40 cm), (5 cm, 20 cm), (5 cm, 20 cm), (15 cm, 40 cm), and (7 cm, 11 cm), respectively. In addition, when the heights were0.6m and 1.2mrespectively, the error distribution of IGWO-ElmanNN was also (0 cm, 4 cm), and the error value with the highest frequency was notably lower than that of the comparison method. This might be because the IGWO algorithm was introduced into the Elman NN, which optimized the initial weights and threshold parameters of the network, thereby improving the model’s search for the global optimal solution, enhancing positioning accuracy, and reducing errors. All error differences have a significance of *p* < 0.05, indicating that these differences are statistically significant. The dynamic analysis results of different methods are shown in Fig 11.

From Fig 11 (a), in comparison with the real trajectory, the IGWO-ElmanNN had a maximum error of about 11 cm and mainly appeared in the topmost trajectory. Based on Fig 11 (b), 11 (c), 11 (d), 11 (e), and 11 (f), in the comparison with the real trajectory, the maximum errors of Elman, PSO-Elman, FA-Elman, BiLSTM NN, and GWO-BiLSTM were 27 cm, 23 cm, 21 cm, 25 cm, and 18 cm, respectively, all of which were greater than 11 cm. This indicated that the IGWO Elman NN had better trajectory fitting ability in dynamic positioning, and its error control was significantly better than the compared algorithms. Moreover, its advantage lied in the optimization of Elman network parameters by the IGWO algorithm, which enabled the model to more accurately capture dynamic signal features and reduce motion trajectory prediction bias. All error differences have a significance of *p* < 0.05, indicating that these differences are statistically significant. The average error comparison of different models along the trajectory is shown in Table 7.

**Table 7:** Comparison of average errors along trajectories for different models.

Algorithm	X/m					Y/m				
	Number of experiments					Number of experiments				
	1	2	3	4	5	1	2	3	4	5
Elman	18.5	18.0	17.8	18.3	18.4	17.7	18.1	18.0	17.9	18.2
PSO-Elman	14.3	14.0	13.9	14.2	14.1	13.8	14.0	13.8	13.0	13.7
FA-Elman	12.7	12.4	12.3	12.6	12.5	12.1	12.2	12.3	12.1	12.1
BiLSTM	16.2	16.3	16.8	16.7	16.8	16.2	16.5	16.6	16.3	16.4
GWO-BiLSTM	9.6	9.4	9.2	9.5	9.3	9.1	9.4	9.3	9.2	9.3
IGWO-Elman	5.2	5.0	4.1	5.1	5.0	4.6	5.0	4.5	5.0	4.8

**Fig 12:**
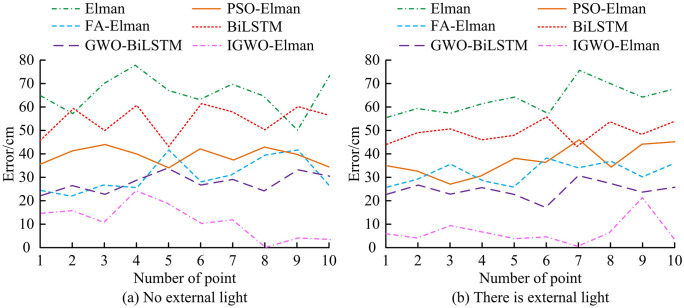
Comparison of environmental light effects of various algorithms.

From Table 7, it can be seen that in the 5 experiments in the X direction, the average error of IGWO-Elman is between 4.1 cm-5.2 cm, while the average error of the comparative algorithm is above 9.0 cm. In the 5 experiments in the Y direction, IGWO-Elman still had the smaller average error, with a range of 4.5 cm-5.0 cm. Next is GWO BiLSTM, with an average error range of 9.1 cm-9.4 cm, while the average error of the remaining comparison algorithms is above 12 cm. Overall, the average error of IGWO-Elman in both X and Y directions is significantly lower than other compared algorithms, fully demonstrating the accuracy advantage of the IGWO-Elman designed in dynamic trajectory positioning. The comparison of the environmental light impact results of various algorithms is shown in Fig 12.

From Fig 12 (a), in the absence of external light, the maximum error of the IGWO-ElmanNN at the selected 10 points was 23.87 cm, which was significantly better than the comparative algorithm. From Fig 12 (b), in the presence of external light, the maximum errors of the IGWO-ElmanNN and the five comparison methods were 21.09 cm, 76.19 cm, 46.55 cm, 38.18 cm, 55.98 cm, and 30.16 cm. The maximum error of the IGWO-ElmanNN in the absence andpresenceof external light was notably smaller than that of the comparison method, indicating that this method had stronger stability and better position perception performance. The core reason was that Resnet50 reduced the interference of ambient light on image data through residual structure and multi-scale feature fusion, and the Elman NN enhanced the robustness of the model to lighting fluctuations by optimizing initial weights and threshold parameters. All error differences have a significance of *p* < 0.05, indicating that these differences are statistically significant.

To further validate the performance of the indoor position perception model in the research design, a certain multi-story building was selected for the study. The multi-story building is an experimental building of a certain university, with a total construction area of approximately 8000 square meters and a total of 5 floors, each with an area of approximately 1600 square meters. In addition, according to the size and function of the room, 4–12 LED lights were arranged in each room to ensure uniform lighting and meet positioning requirements. When applying the model to this multi-story building, localization settings were required. This was because there were differences in building structure, environmental light interference, and personnel activities, such as the layout of different buildings, wall materials, floor heights, and other structural factors that can have different effects on the propagation of visible light signals [39,40]. By localizing settings, the layout of light sources and signal acquisition methods could be optimized based on the specific structural characteristics of the experimental building, thereby improving positioning accuracy. Therefore, the study conducted localized configuration in three aspects, namely light source optimization adjustment, data acquisition optimization, and model parameter adjustment. Specifically, in terms of adjustment methods, multi-objective optimization based on lighting uniformity and positioning error was conducted through field testing and DIALux Evo simulation. The number of LED lights in areas with frequent personnel flow such as corridors and stairwells is increased, and their installation angles are adjusted, with 6 LEDs per laboratory, 2 × 3 arrays, installation height of 3m, spacing of 1.5m, total power of 32W, average illumination of 200lux, and uniformity of 88.5%. During the configuration determination process, the study conducted simulations and field tests on 5 LED configurations, totaling 4–8, and ultimately selected 6 LEDs due to their minimal positioning error. Meanwhile, representative locations such as laboratories, classrooms, and offices were selected for data collection, and based on these data, the number of hidden layer nodes in the indoor location perception model was 120, with a learning rate of 0.001. In terms of positioning technology, the research has incorporated Bluetooth positioning, wireless local area network positioning, and ultra wideband positioning, as well as the passive radio frequency identification positioning method designed by experts such as Xiao F, which integrated received signal strength indicators and phase measurements, and the three-dimensional passive radio frequency indoor positioning technology based on received signal strength distribution and Gaussian process regression designed by Yuan L et al [41,42]. In addition, the study also introduced the improved Northern Eagle algorithm based on received signal strength ratio designed by Zhang H et al. to optimize the Elman neural network for indoor visible light positioning system [43]. The comparison of positioning errors and accuracy of different positioning technologies on multi-story buildings is shown in Table 8.

**Table 8:** Comparison of positioning errors and accuracy of different positioning technologies on multi story buildings.

Technology	Root Mean Square Error (RMSE)/m				Positioning accuracy/%			
	Number of experiments				Number of experiments			
	1	2	3	4	1	2	3	4
Bluetooth positioning	0.994	1.039	1.026	0.910	84.10	85.16	84.31	86.96
Wireless local area networkspositioning	0.894	0.812	0.837	0.815	86.76	87.74	86.19	86.55
Ultra wideband positioning	0.668	0.771	0.761	0.733	87.93	87.34	87.63	88.03
Xiao F et al	0.344	0.352	0.296	0.334	94.86	94.54	94.06	94.12
Yuan L et al	0.292	0.231	0.287	0.290	95.69	95.62	95.03	95.07
Zhang H et al	0.007	0.014	0.015	0.008	98.21	99.03	99.14	99.15
IGWO-Elman	0.002	0.006	0.012	0.005	99.75	99.45	99.62	99.50

From Table 8, different positioning techniques had significant differences in positioning errors and accuracy on multi story buildings. The IGWO Elman positioning technology designed for research had significant advantages, with RMSE far lower than other technologies in all four experiments, at 0.002m, 0.006m, 0.012m, and 0.005m, respectively, and positioning accuracy exceeding 99%. The RMSE of Bluetooth positioning fluctuated greatly, with an accuracy of only 84.10% −86.96%. All differences have a significance of *p* < 0.05, indicating that these differences are statistically significant. IGWO Elman positioning technology has extremely low root mean square error and over 99% accuracy, because the multi story building is a university experimental building, the experimental area is a 3-story laboratory, and each floor has no complex obstructions. The LED layout has also been localized and optimized, and a light uniformity of 91.2%. The experiment was conducted at night with minimal environmental interference, close to a controlled experimental environment, and different from “large-scale complex environments”, resulting in lower errors. The improved Northern Eagle algorithm based on received signal strength ratio designed by Zhang H et al. optimizes the Elman neural network for indoor positioning system. Although it has achieved some improvement in positioning accuracy, it still has certain limitations in environmental adaptability and dynamic performance. Compared with the refractive reverse learning strategy used to initialize the population in Zhang H. et al.‘s [43] improved Northern Eagle algorithm, IGWO adopts an adaptive initialization method based on dynamic environment perception, which can automatically adjust the initial population according to real-time environmental changes, thus better adapting to dynamic environments. Compared with the non-linear weight factors introduced in the improved Northern Eagle algorithm to accelerate convergence speed, IGWO adopts an adaptive weight adjustment mechanism to dynamically adjust weights based on the search state during the iteration process, which not only accelerates convergence speed but also improves search accuracy. In addition, in terms of positioning error during LED emission power fluctuations, IGWO can maintain lower positioning error under the same conditions and demonstrate stronger anti-interference ability through enhanced robustness design. These improvements make IGWO superior to the improved Northern Eagle algorithm in terms of positioning accuracy, convergence speed, and robustness, making it more suitable for complex indoor positioning scenarios. And the RMSE and accuracy performance of wireless local area networks positioning and ultra wideband positioning were also inferior to the technology studied and designed. To investigate the reasons, the feature extraction method based on Resnet50 in research and design positioning technology could efficiently obtain image depth features, and it combined with IGWO optimized Elman NN to accurately perceive position. Meanwhile, the localization of the experimental building, such as optimizing the layout of light sources, data acquisition, and adjusting model parameters, further enhanced the adaptability and robustness of the model, enabling it to maintain high accuracy and stability in complex environments.

The analysis of the example results has verified the positioning ability of the proposed method under multiple heights, dynamic environments, and different lighting conditions through different experimental scenarios. The results showed that the proposed method had higher accuracy and stronger environmental adaptability compared to traditional methods. Through case studies in multi-story buildings, the advantages of the proposed method in practical applications were further demonstrated, including lower positioning errors and higher positioning accuracy, which also proved its effectiveness and practicality in actual indoor positioning problems.

Although this work assumed that the visual sensor needs to be within the line of sight of the LED, slight directional errors in the visual sensor may introduce certain errors. However, due to the robustness of the Resnet50 feature extraction designed for research and the Elman NN optimized based on IGWO to slight directional errors in visual sensors, which can reduce their impact on position prediction accuracy, the impact of this error was not significant. In order to quantify and validate the robustness of visual sensors under slight directional errors, the performance of the model was evaluated by measuring the positioning accuracy under different directional deviations. The experimental verification of slight directional errors is shown in Table 9.

**Table 9:** Experimental verification of slight directional errors.

Direction deviation angle/°	Sample size	Average positioning error/cm	Maximum positioning error/cm	Minimum positioning error/cm	Standard deviation/cm
0	100	0.85	1.20	0.5	0.20
5	100	0.92	1.35	0.6	0.25
10	100	1.05	1.50	0.7	0.30
15	100	1.20	1.75	0.8	0.35
20	100	1.35	2.00	0.9	0.40

From Table 9, it can be seen that as the directional deviation of the visual sensor increases from 0° to 20°, the average positioning error increases from 0.85 cm to 1.35 cm, the maximum positioning error increases from 1.20 cm to 2.00 cm, the minimum positioning error increases from 0.50 cm to 0.90 cm, and the standard deviation increases from 0.20 cm to 0.40 cm. Although the error gradually increases with the increase of directional deviation, the overall error remains at a low level, indicating that the proposed model has good robustness in the face of slight directional errors. Especially under directional deviations within 10°, the increase in average positioning error is only 0.20 cm, and the change in standard deviation is small, further proving the positioning accuracy and stability of the model under different directional deviations, providing strong theoretical support for small sensor directional deviations in practical applications. The computational cost and real-time performance of the entire indoor position perception process are shown in Table 10.

**Table 10:** The computational cost and real-time performance of the entire indoor position perception process.

Process	FLOPs	Single sample time/ms	Proportion of total time spent/%	Hardware resource utilization (peak)
Image acquisition	\	33.3	40.07%	Central Processing Unit (CPU) 15%, memory 1.2GB
Bilinear interpolation preprocessing	8.2 × 10^6^	4.7	5.66%	CPU 30%, memory 1.5GB
ResNet50 feature extraction	4.1 × 10^9^	43.0	51.74%	CPU 65%, memory 2.5GB
IGWO Elman position estimation	1.2 × 10^6^	2.1	2.53%	CPU 40%, memory 2.0GB
Total	4.1 × 10^9^ + 9.4 × 10^6^	83.1	100%	CPU 65%, memory 2.5GB

From Table 10, it can be seen that in the entire indoor position perception process, the FLOPs extracted by ResNet50 feature extraction reached 4.1 × 10^9^, with a single sample time of 43.0ms, accounting for 51.74% of the total time, which is the main part of the computational cost. The computational complexity of bilinear interpolation preprocessing and IGWO Elman position estimation is relatively small, with single sample times of 4.7ms and 2.1ms, respectively, and a lower time proportion. Although there is no FLOPs data available for image acquisition, a single sample takes 33.3ms and accounts for 40.07% of the total time. The utilization of hardware resources in each link is within a reasonable range, and the total single sample time is 83.1ms. Overall, the indoor location sensing process has good real-time performance and cost control effect.

## 5. Discussion

The deep feature extraction and IGWO optimization model based on Resnet50 proposed in this article has the potential for cross scene applications in the field of visual perception. For example, in the field of building health monitoring, we can draw on the research ideas of Hu K et al., which aims to address the problem of difficulty in obtaining three-dimensional information of cracks from two-dimensional images. A three-dimensional detection system is built through multi-sensor fusion, and crack localization is achieved through sensor calibration and semantic segmentation. Then, sub millimeter level precision three-dimensional crack features are obtained by relying on robotic arms and depth cameras, providing a solution for automated monitoring of structural health [44]. Meanwhile, in machine learning driven industrial inspection scenarios, the model optimization approach proposed in this paper can be further expanded. For example, experts such as Yao M have solved the problem of detecting debonding defects in concrete filled steel tube arch bridges by combining Bayesian optimization and long short-term memory networks to solve the traditional parameter optimization problem, improving prediction accuracy up to [45]. This article’s model can draw on this idea and apply the deep feature extraction capability of Resnet50 to defect feature mining in ultrasound detection data, replacing traditional manual feature screening. Combined with the IGWO optimization algorithm, the parameters of the detection model are optimized to further improve the accuracy and efficiency of debonding defect recognition, providing technical support for machine learning driven industrial detection scenarios.

## 6. Conclusion and future work

To improve the stability and accuracy of the received signal strength perception technology, a feature extraction method based on Resnet50 was studied and designed, and an indoor position perception model based on IGWO-ElmanNN was constructed. The results showed that in the light source layout, the four LED layout performed better, with a corresponding uniformity of 91.21%, notably better than the comparative layout. The feature extraction method designed in the research could extract more diverse and repetitive features, and its average time was 43ms, notably lower than the comparison method. Under different test functions, the average fitness value and average standard deviation of the IGWO algorithm were notably lower than those of the comparison algorithm. For example, on the unimodal test functionf2, the average standard deviations of IGWO and the comparison algorithm were 6.31 × 10^3^, 8.15 × 10^3^, 8.74 × 10^3^, 9.56 × 10^3^, and 7.60 × 10^3^, respectively. This indicated that the IGWO algorithm could more effectively explore the search space and find points closer to the global optimal solution. When the heights were 0m, 0.6m, and 1.2m respectively, the error of IGWO-ElmanNNwas smaller. This may be because IGWO’s parameter optimization of Elman NNs avoided the problem of easily falling into local optima, thereby improving the model’s global search ability and convergence speed, enabling IGWO-ElmanNNs to more accurately perceive indoor positions at different heights. In terms of method stability, the maximum errors ofIGWO-ElmanNN in the absence and presence of external light were23.87 cm and 21.09 cm,respectively, which were notably lower than the comparative methods. Overall, the designed indoor position perception model had good stability and accuracy.

## 7. Limitation and future work

The study also has certain limitations. Manual data collection requires operators to hold visual sensors and collect data point by point at preset locations, which is highly time-consuming and difficult to meet the demand for rapid data acquisition in large-scale indoor spaces. Additionally, due to variations in human operation, the quality of collected data fluctuates significantly, resulting in unstable data quality. Moreover, large-scale data collection requires multiple personnel collaboration, and operators need training, leading to high training and labor costs. Future research will focus on improving the automation of data collection, enhancing data objectivity and efficiency. For example, developing an automated data collection system based on mobile robots, integrating visual sensors and inertial measurement units, to achieve autonomous navigation and image acquisition in indoor spaces. This will address the inefficiency and susceptibility to human interference in manual collection while expanding dataset scale through multi-device collaborative acquisition, thereby improving model generalization capabilities.

The experiment assumes that the visual sensor must remain within the LED’s line of sight. Slight deviations in sensor orientation may affect positioning accuracy, although the proposed method exhibits a certain degree of robustness against such errors. Additionally, this study was primarily conducted under specific experimental conditions. While localization settings enhanced the model’s adaptability and robustness, factors such as architectural structural variations, ambient light interference, and human activity may still impact positioning performance in broader real-world applications. Future research could optimize the layout and calibration of visual sensors through machine learning-based sensor calibration algorithms, employ environmental perception technologies like light intensity sensors and ambient spectral analysis to monitor and compensate for ambient light interference in real time, and further refine the model using deep learning techniques.

Additionally, the primary reason for the maximum error of IGWO-Elman increasing to 21.09 cm under external lighting is the interference of ambient light on the LED spot characteristics, which reduces the grayscale contrast of LED spots in the image. Consequently, the recognition accuracy of features such as spot edges and grayscale gradients extracted by ResNet50 declines, and the proportion of similar features in the feature database increases, ultimately leading to larger positioning errors. This also highlights the limitations of the feature extraction method in the research design when dealing with environmental noise, namely insufficient robustness against spectral interference and a lack of dynamic lighting adaptation capability. Therefore, future research will explore more advanced algorithms to enhance the model’s adaptability and robustness in dynamic and complex environments. Specifically, efforts will focus on improving the ResNet50-based feature extraction network with attention mechanisms to strengthen feature focusing on LED light source regions and reduce interference from ambient light and dynamic obstacles in feature extraction. Simultaneously, reinforcement learning algorithms will be introduced to optimize the IGWO search strategy, further balancing global and local search capabilities, avoiding algorithmic convergence to local optima, and improving the model’s positioning stability in complex dynamic environments. Additionally, integrating visible light image sensors with Bluetooth and ultra-wideband sensor data will establish a multimodal feature fusion framework, leveraging the high precision of visible light with the non-line-of-sight positioning capabilities of Bluetooth/ultra-wideband to overcome the constraint of “visual sensors requiring line-of-sight with LEDs,” achieving full-scenario indoor positioning coverage.

## Supporting information

S1 File Minimal data set definition. (DOC)
