# Supplementary material for: Indoor location perception model based on Resnet50 and Elman network
Source: PLoS One. 2025 Dec 22;20(12):e0338316. doi: 10.1371/journal.pone.0338316 (PMC12721536; doi:10.1371/journal.pone.0338316)
Supplement: S1 File — (DOC) [file pone.0338316.s001.doc]

**Minimal Data Set Definition**

**The data in Figure 8**

| (a) Comparison of total power | | (b) Comparison of illuminance | | (c) Comparison of uniformity | |
| --- | --- | --- | --- | --- | --- |
| Layout situation | Value/W | Layout situation | Value/W | Layout situation | Value/W |
| 3 LEDs | 175 | 3 LEDs | 668.951 | 3 LEDs | 75.6% |
| 4 LEDs | 175 | 4 LEDs | 423.661 | 4 LEDs | 91.21% |
| 5 LEDs | 152 | 5 LEDs | 377.481 | 5 LEDs | 91.22% |

The data in Figure 9

| Algorithm | (a) Unimodal function f2 (×10^5^) | | | | | | | | | |
| --- | --- | --- | --- | --- | --- | --- | --- | --- | --- | --- |
|  | Quantity of trials | | | | | | | | | |
|  | 5 | 10 | 15 | 20 | 25 | 30 | 35 | 40 | 45 | 50 |
| PSO | 10.56 | 8.74 | 10.14 | 9.24 | 10.14 | 9.07 | 10.31 | 8.74 | 10.06 | 9.15 |
| GA | 13.20 | 9.65 | 11.38 | 10.89 | 12.04 | 9.90 | 11.46 | 10.89 | 10.39 | 9.73 |
| GWO | 11.88 | 12.78 | 11.88 | 10.05 | 10.72 | 10.56 | 10.89 | 8.41 | 10.72 | 11.55 |
| Combination algorithm 1 | 6.02 | 7.75 | 8.08 | 7.18 | 7.92 | 7.92 | 7.92 | 7.59 | 7.75 | 7.09 |
| IGWO (manuscript) | 2.31 | 2.72 | 2.56 | 2.56 | 2.64 | 2.31 | 2.64 | 2.31 | 2.64 | 2.47 |
| Algorithm | (b) Multi peak function f9 (×10^1^) | | | | | | | | | |
|  | Quantity of trials | | | | | | | | | |
|  | 5 | 10 | 15 | 20 | 25 | 30 | 35 | 40 | 45 | 50 |
| PSO | 7.67 | 4.45 | 5.20 | 6.76 | 6.19 | 6.93 | 5.86 | 7.18 | 6.52 | 5.61 |
| GA | 8.99 | 6.60 | 7.18 | 7.75 | 6.85 | 7.92 | 5.28 | 7.59 | 5.77 | 7.01 |
| GWO | 9.65 | 8.25 | 9.24 | 5.20 | 9.98 | 5.94 | 8.49 | 7.92 | 8.49 | 7.75 |
| Combination algorithm 1 | 3.30 | 3.96 | 4.04 | 4.04 | 4.37 | 3.46 | 3.13 | 4.54 | 3.05 | 6.02 |
| IGWO (manuscript) | 1.15 | 0.99 | 1.65 | 1.81 | 1.24 | 1.32 | 1.40 | 1.40 | 0.91 | 1.07 |
| Algorithm | (c) Mixed function f24 (×10^2^) | | | | | | | | | |
|  | Quantity of trials | | | | | | | | | |
|  | 5 | 10 | 15 | 20 | 25 | 30 | 35 | 40 | 45 | 50 |
| PSO | 4.95 | 3.79 | 3.71 | 7.75 | 4.21 | 4.95 | 2.64 | 5.69 | 5.44 | 4.78 |
| GA | 5.86 | 7.18 | 4.70 | 4.04 | 5.44 | 6.76 | 6.19 | 4.12 | 5.94 | 3.79 |
| GWO | 8.49 | 8.00 | 6.93 | 7.09 | 6.52 | 5.94 | 5.77 | 6.02 | 6.19 | 6.60 |
| Combination algorithm 1 | 4.54 | 9.40 | 6.10 | 4.78 | 5.77 | 7.34 | 5.20 | 6.35 | 7.42 | 5.61 |
| IGWO (manuscript) | 1.65 | 1.65 | 3.05 | 1.81 | 1.90 | 2.56 | 2.06 | 2.64 | 2.14 | 1.40 |

**The data in Figure 10**

| Algorithm | (a) Height=0m | | | | | | | | | |
| --- | --- | --- | --- | --- | --- | --- | --- | --- | --- | --- |
|  | Quantity of trials | | | | | | | | | |
|  | 5 | 10 | 15 | 20 | 25 | 30 | 35 | 40 | 45 | 50 |
| Elman | 36.67 | 32.31 | 32.05 | 33.59 | 23.59 | 27.69 | 34.36 | 33.33 | 30.51 | 31.54 |
| PSO-Elman | 17.95 | 21.28 | 18.72 | 13.33 | 15.13 | 13.59 | 16.92 | 14.10 | 12.05 | 10.51 |
| FA-Elman | 11.54 | 14.36 | 12.56 | 15.90 | 11.28 | 10.77 | 13.59 | 16.15 | 9.49 | 12.82 |
| BiLSTM | 30.26 | 27.69 | 30.51 | 35.90 | 38.46 | 36.92 | 31.03 | 27.44 | 33.08 | 34.87 |
| GWO-BiLSTM | 8.46 | 10.26 | 10.77 | 7.44 | 5.90 | 8.97 | 9.74 | 10.26 | 7.95 | 7.95 |
| IGWO-Elman | 5.90 | 2.31 | 2.05 | 3.59 | 0.77 | 2.82 | 3.85 | 4.10 | 2.56 | 3.33 |
| Algorithm | (b) Height=0.6m | | | | | | | | | |
|  | Quantity of trials | | | | | | | | | |
|  | 5 | 10 | 15 | 20 | 25 | 30 | 35 | 40 | 45 | 50 |
| Elman | 29.23 | 34.62 | 31.03 | 31.28 | 28.72 | 36.92 | 29.49 | 27.95 | 29.74 | 37.44 |
| PSO-Elman | 14.36 | 17.69 | 18.46 | 14.10 | 17.95 | 17.18 | 13.59 | 14.62 | 16.92 | 16.15 |
| FA-Elman | 16.92 | 14.62 | 8.97 | 11.79 | 15.38 | 22.05 | 11.28 | 16.92 | 19.74 | 12.05 |
| BiLSTM | 45.64 | 21.79 | 41.54 | 37.44 | 33.59 | 33.85 | 31.79 | 35.90 | 33.08 | 26.15 |
| GWO-BiLSTM | 11.54 | 7.18 | 11.79 | 8.72 | 7.44 | 9.49 | 10.00 | 7.18 | 8.72 | 10.26 |
| IGWO-Elman | 2.82 | 2.05 | 0.51 | 2.56 | 4.62 | 4.10 | 3.08 | 1.79 | 1.79 | 2.31 |
| Algorithm | (c) Height=1.2m | | | | | | | | | |
|  | Quantity of trials | | | | | | | | | |
|  | 5 | 10 | 15 | 20 | 25 | 30 | 35 | 40 | 45 | 50 |
| Elman | 35.13 | 37.18 | 40.00 | 32.56 | 18.72 | 20.77 | 25.64 | 24.36 | 47.69 | 38.21 |
| PSO-Elman | 15.90 | 13.59 | 9.49 | 24.87 | 14.62 | 12.56 | 17.69 | 14.87 | 16.15 | 13.85 |
| FA-Elman | 13.59 | 16.15 | 21.79 | 16.41 | 16.67 | 24.10 | 7.44 | 12.05 | 13.59 | 27.44 |
| BiLSTM | 38.72 | 34.10 | 36.15 | 35.13 | 40.51 | 35.90 | 34.36 | 30.77 | 30.51 | 36.41 |
| GWO-BiLSTM | 9.49 | 7.44 | 7.18 | 11.79 | 9.74 | 8.21 | 9.49 | 9.23 | 7.95 | 6.92 |
| IGWO-Elman | 2.56 | 4.10 | 3.08 | 2.31 | 1.79 | 3.85 | 3.08 | 2.31 | 2.31 | 3.33 |

**The data in Figure 12**

| Algorithm | (a) No external light | | | | | | | | | |
| --- | --- | --- | --- | --- | --- | --- | --- | --- | --- | --- |
|  | Number of point | | | | | | | | | |
|  | 1 | 2 | 3 | 4 | 5 | 6 | 7 | 8 | 9 | 10 |
| Elman | 65.15 | 56.91 | 70.10 | 77.11 | 67.22 | 63.09 | 69.69 | 65.57 | 50.31 | 73.40 |
| PSO-Elman | 35.88 | 40.82 | 43.71 | 39.59 | 34.23 | 42.06 | 37.53 | 43.30 | 39.59 | 34.64 |
| FA-Elman | 24.74 | 21.44 | 26.80 | 25.15 | 41.24 | 28.04 | 31.34 | 38.76 | 41.24 | 26.80 |
| BiLSTM | 46.19 | 59.38 | 50.31 | 60.62 | 42.89 | 61.44 | 57.73 | 50.72 | 59.79 | 56.91 |
| GWO-BiLSTM | 21.86 | 26.39 | 22.68 | 28.45 | 33.81 | 26.80 | 28.87 | 24.33 | 32.99 | 30.52 |
| IGWO-Elman | 14.85 | 15.26 | 10.72 | 23.51 | 18.97 | 10.31 | 11.55 | 0.41 | 4.12 | 3.30 |
| Algorithm | (b) There is external light | | | | | | | | | |
|  | Number of point | | | | | | | | | |
|  | 1 | 2 | 3 | 4 | 5 | 6 | 7 | 8 | 9 | 10 |
| Elman | 56.08 | 59.38 | 57.73 | 61.44 | 63.92 | 56.91 | 75.46 | 70.10 | 63.92 | 67.63 |
| PSO-Elman | 34.64 | 32.58 | 27.22 | 30.93 | 37.94 | 36.29 | 46.19 | 34.64 | 44.12 | 44.54 |
| FA-Elman | 25.98 | 29.28 | 35.46 | 28.87 | 25.57 | 37.94 | 34.23 | 37.11 | 30.52 | 35.46 |
| BiLSTM | 44.12 | 48.66 | 50.31 | 46.19 | 47.42 | 55.67 | 42.89 | 53.61 | 48.25 | 53.20 |
| GWO-BiLSTM | 22.27 | 26.39 | 22.27 | 25.15 | 22.68 | 17.32 | 30.52 | 27.22 | 23.92 | 25.15 |
| IGWO-Elman | 5.36 | 3.71 | 9.07 | 6.60 | 4.12 | 4.54 | 0.82 | 6.19 | 21.44 | 4.12 |
